# Preparation of Zr(Mo,W)_2_O_8_ with a larger negative thermal expansion by controlling the thermal decomposition of Zr(Mo,W)_2_(OH,Cl)_2_∙2H_2_O

**DOI:** 10.1038/s41598-018-23529-6

**Published:** 2018-03-28

**Authors:** Mariya Yu. Petrushina, Elena S. Dedova, Eugeny Yu. Filatov, Pavel E. Plyusnin, Sergei V. Korenev, Sergei N. Kulkov, Elizaveta A. Derevyannikova, Marat R. Sharafutdinov, Alexander I. Gubanov

**Affiliations:** 1 Nikolaev Institute of Inorganic Chemistry, Siberian Branch of the Russian Academy of Sciences, Academician Lavrentiev Prospekt 3, 630090 Novosibirsk, Russian Federation; 2 Novosibirsk State University, Pirogova str. 2, 630090 Novosibirsk, Russian Federation; 3 Institute of Strength Physics and Materials Science, Siberian Branch of the Russian Academy of Sciences, pr. Academicheskii 2/4, 634021 Tomsk, Russian Federation; 4 Tomsk Polytechnic University, Lenin Avenue 30, 634050 Tomsk, Russian Federation; 5 Boreskov Institute of Catalysis, Siberian Branch of the Russian Academy of Sciences, Acad. Lavrentiev Prospekt 5, 630090 Novosibirsk, Russian Federation; 6 Institute of Solid State Chemistry and Mechanochemistry of the Siberian Branch of the Russian Academy of Sciences, Kutateladze 18, 630128 Novosibirsk, Russian Federation; 7 Budker Institute of Nuclear Physics of the Siberian Branch of the Russian Academy of Sciences, Acad. Lavrentiev Prospekt 11, 630090 Novosibirsk, Russian Federation

## Abstract

Solid solutions of Zr(Mo,W)_2_O_7_(OH,Cl)_2_∙2H_2_O with a preset ratio of components were prepared by a hydrothermal method. The chemical composition of the solutions was determined by energy dispersive X-ray spectroscopy (EDX). For all the samples of ZrMo_x_W_2−x_O_7_(OH,Cl)_2_∙2H_2_O (x = 0.0, 0.2, 0.4, 0.6, 0.8, 1.0, 1.2, 1.4, 1.6, 1.8, and 2.0), TGA and *in situ* powder X-ray diffraction (PXRD) studies (300–1100 K) were conducted. For each case, the boundaries of the transformations were determined: Zr(Mo,W)_2_O_7_(OH,Cl)_2_∙2H_2_O → orthorhombic-ZrMo_x_W_2−x_O_8_ (425–525 K), orthorhombic-ZrMo_x_W_2−x_O_8_ → cubic-ZrMo_x_W_2−x_O_8_ (700–850 K), cubic-ZrMo_x_W_2−x_O_8_ → trigonal-ZrMo_x_W_2−x_O_8_ (800–1050 K for x > 1) and cubic-ZrMo_x_W_2−x_O_8_ → oxides (1000–1075 K for x ≤ 1). The cell parameters of the disordered cubic-ZrMo_x_W_2−x_O_8_ (space group Pa-3) were measured within 300–900 K, and the thermal expansion coefficients were calculated: −3.5∙10^−6^ – −4.5∙10^−6^ K^−1^. For the ordered ZrMo_1.8_W_0.2_O_8_ (space group P2_1_3), a negative thermal expansion (NTE) coefficient −9.6∙10^−6^ K^−1^ (300-400 K) was calculated. Orthorhombic-ZrW2O_8_ is formed upon the decomposition of ZrW_2_O_7_(OH,Cl)_2_∙2H_2_O within 500–800 K.

## Introduction

Both applied material science and academic research fields are interested in substances with negative thermal expansion (NTE)^1–4^. Due to its unique behaviour, ZrMo_x_W_2−x_O_8_ is an advanced filler material used to produce composites with controlled expansion. The main advantage of the family of cubic-ZrMo_x_W_2−x_O_8_ materials over other materials is their large isotropic NTE over a wide temperature range. The most famous and well-studied ZrW_2_O_8_ has an NTE coefficient of −8.8∙10^−6^ K^−1^ for oxygen-ordered cubic α-ZrW_2_O_8_ from 0.3 K to 430 K and −4.8∙10^−6^ K^−1^ for dynamically disordered cubic β-ZrW_2_O_8_ from 430 K to 1050 K^5^. Cubic γ-ZrMo_2_O_8_ is isostructural to cubic β-ZrW_2_O_8_, and it has an NTE coefficient of −5.0∙10^-6^ K^−1^ from 11 to 573 K, as reported in ref.^6^. Simon Allen and J.S.O. Evans made precise measurements of the NTE coefficient of γ-ZrMo_2_O_8_: −6.9∙10^−6^ K^−1^ from 2 to 200 K and −5.0∙10^−6^ K^−1^ from 250 to 502 K^7^.

This material can be obtained by dehydration and topotactic recrystallization from a hydrated precursor, ZrMo_x_W_2−x_(OH,Cl)_2_∙2H_2_O^8^. According to the thermogravimetric results presented by us in a previous paper^9^, the thermal decomposition of the precursor ZrW_2_O_7_(OH,Cl)_2_∙2H_2_O starts at 500 K and results in the appearance of an amorphous intermediate, which crystallizes as cubic β-ZrW_2_O_8_ above 850 K. The strong exothermic effect at 1100 K is related to the decomposition of cubic β-ZrW_2_O_8_ into oxides. The transformation of cubic α-ZrW_2_O_8_ into cubic β-ZrW_2_O_8_ is not registered by differential thermal analysis (DTA).

The DTA curve for ZrMo_x_W_2−x_O_7_(OH,Cl)_2_∙2H_2_O showed the expected endothermic peak, corresponding to the weight loss and formation of the orthorhombic phase of β-ZrMo_2_O_8_, while the two exothermic peaks at 700 and 800 K can be assigned to the crystallization of the cubic γ-ZrMo_2_O_8_ and trigonal α−ZrMo_2_O_8_ phases, respectively^10^.

The solid solutions of ZrW_2−x_Mo_x_O_8_ (with different values of “*x*”) have been prepared by a number of scientific groups^7,8,11–14^. A significant number of solid solutions of the precursor ZrMo_x_W_2−x_O_7_(OH)_2_∙2H_2_O was prepared by Ling Huang and co-authors^12^. Cubic phases were obtained for x = 0.2, 0.4, 0.6, 0.7 and 0.8, but the NTE coefficients were not measured. Accurate measurement of the NTE coefficient for ZrMo_x_W_2−x_O_8_ (x = 1.0) was reported in work^6^. For the cubic, ordered phase, the NTE was −9.0∙10^−6^ K^−1^ from 2 to 200 K, and −5.5∙10^−6^ K^−1^ from 250 to 502 K for the disordered phase. Solid solutions of ZrMo_x_W_2−x_O_8_ (x = 0.4, 0.6, 0.7, 1.0 1.2, 1.4 and 1.5) were prepared by C. Closmann^8^. The phase composition was determined for all the samples after heating them to 723, 823, and 923 K and quenching. For one of these solutions (ZrMo_0__.__4_W_1.6_O_8_), the NTE coefficient was −11.8∙10^−6^ K^−1^ (273-298 K) and –7.4∙10^−6^ K^−1^ (383–473 K). The NTE coefficient –11.8∙10^–6^ K^−1^ is the largest known (in absolute value).

We assumed that one of the cubic-ZrMo_x_W_2−x_O_8_ solutions may have a larger coefficient than that previously determined. We carefully investigated the conditions for obtaining cubic-ZrMo_x_W_2−x_O_8_ (x = 0.0, 0.2, 0.4, 0.6, 0.8, 1.0, 1.2, 1.4, 1.6, 1.8, and 2.0) from precursors and measured their NTE coefficients.

## Results and Discussion

### Synthesis of the precursors and their characterization

We synthesized ZrMo_x_W_2−x_O_7_(OH,Cl)_2_∙2H_2_O by a hydrothermal method^10^. Stoichiometric amounts of Na_2_WO_4_•2H_2_O, Na_2_MoO_4_ and ZrOCl_2_•8H_2_O were placed in a flask with 2.3 M HCl (solid: liquid ~1: 10) and stirred for 30 minutes. The obtained reaction mixture was placed in an autoclave with a Teflon liner and heated at 450 K for 48 hours. After cooling, ZrMo_x_W_2−x_O_7_(OH,Cl)_2_∙2H_2_O was filtered, washed with water and dried at 380 K for 24 hours. Eleven samples were obtained. The colours of the powders of ZrMo_x_W_2−x_O_7_(OH,Cl)_2_∙2H_2_O changed from white to bluish as a function of the value of “*x”*.

**Figure 1:**
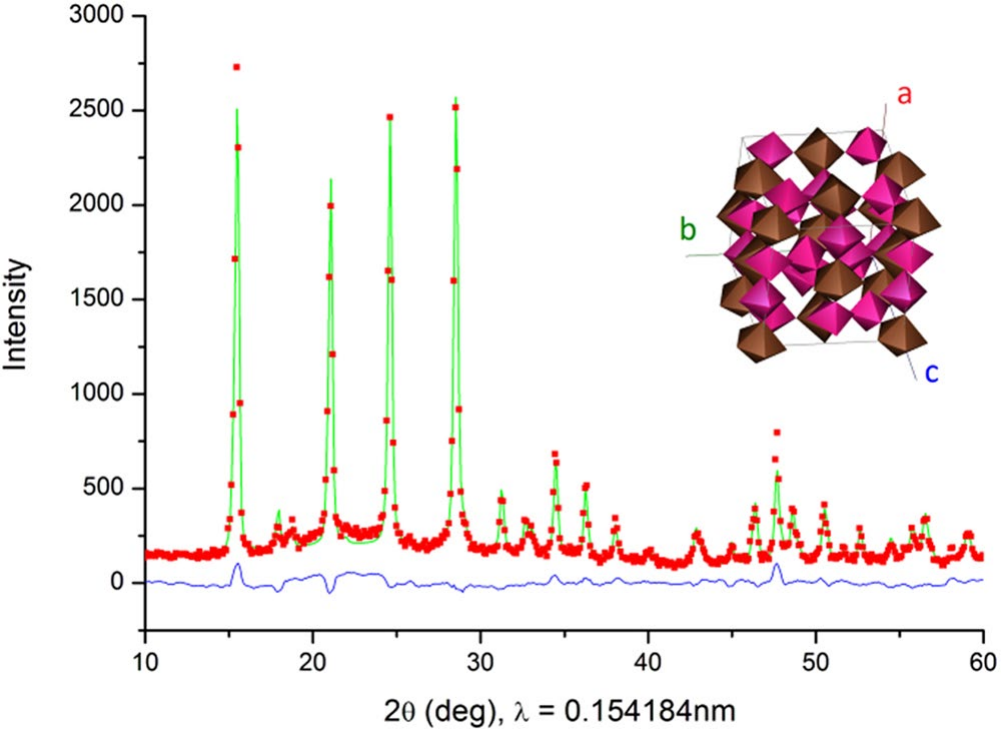
A comparison of the PXRD diffraction pattern (red dots) for ZrMo_x_W_2−x_O_7_(OH,Cl)_2_∙2H_2_O (x = 1.8) with that calculated by the Rietveld refinement model (green solid line). The corresponding crystal structure is shown in the inset (pink octahedra – {W(Mo)O_6_}, brown pentagonal bipyramids – {Zr(Cl,O)_7_}).

The powders of ZrMo_x_W_2−x_O_7_(OH,Cl)_2_∙2H_2_O were examined by powder X-ray diffraction (Fig. 1) on a DRON-RM4 diffractometer (Cu*K*_α_ source, graphite monochromator at the diffracted beam, room temperature, 2θ range 5–60°). The experimental data were processed with the PowderCell program v.2.4^15^, and the data from the powder structural database powder diffraction file (PDF) were used as the reference^16^. The EDX spectral analysis was performed using a Hitachi TM3030 desktop scanning electron microscope and the Quantax70 microanalysis system.

The PXRD data showed that all the samples were single phase. We performed an EDX investigation for all the prepared samples and found that the preset stoichiometry was retained in the products of the reaction within the experimental uncertainty. Determining the content of molybdenum and tungsten in ZrMo_x_W_2−x_O_7_(OH,Cl)_2_∙2H_2_O by the Zen-Retgers rule is extremely difficult because of the extreme similarity of the unit cell volumes (Table 1).

**Table 1:** Data for the EDX experiment for ZrMo_x_W_2−x_O_7_(OH,Cl)_2_∙2H_2_O and the calculated cell parameters (space group I4_1_cd).

x, set value	x, found	a, Å	c	V/Z (Z = 8)
0	0.00	11.461(2)^*^	12.509(2)^*^	205.39(4)^*^
0.2	0.24(2)	11.417(1)	12.454 (1)	202.92(3)
0.4	0.42(2)	11.218 (1)	12.235(1)	192.47(3)
0.6	0.58(2)	11.390(1)	12.414(2)	201.30(5)
0.8	0.80(2)	11.244(1)	12.276(1)	194.01(3)
1.0	1.00(2)	11.439(1)	12.498(1)	204.42(3)
1.2	1.20(2)	11.393(1)	12.429 (1)	201.68(4)
1.4	1.40(2)	11.404 (1)	12.450 (2)	202.37(3)
1.6	1.54(2)	11.384(1)	12.427(2)	201.29(4)
1.8	1.74(2)	11.429(1)	12.471(1)	203.61(4)
2	2.00	11.447(1)	12.495 (1)	204.67(3)

^*^ISCD #157755.

### DTA and high-temperature PXRD of ZrMo_x_W_2–x_O_7_(OH,Cl)_2_∙2H_2_O

A thermal analysis was performed on an “STA 449 F1 Jupiter” in a platinum crucible under an oxygen–argon atmosphere (20% O_2_) in the temperature range 298–1073 K.

High-temperature experiments were carried with time-resolved diffractometry at channel 5b of the Siberian Synchrotron and Therahertz Radiation Centre^17,18^. The wavelength used was 1.516 A. The diffraction patterns were recorded by a one-coordinate detector (OD-3) developed in Budker Institute of Nuclear Physics of Siberian Branch Russian Academy of Sciences^19^. The exposure time for a frame was set to 1 minute. The samples were heated in air up to 1123 K at a rate of 10 K/min.

**Figure 2:**
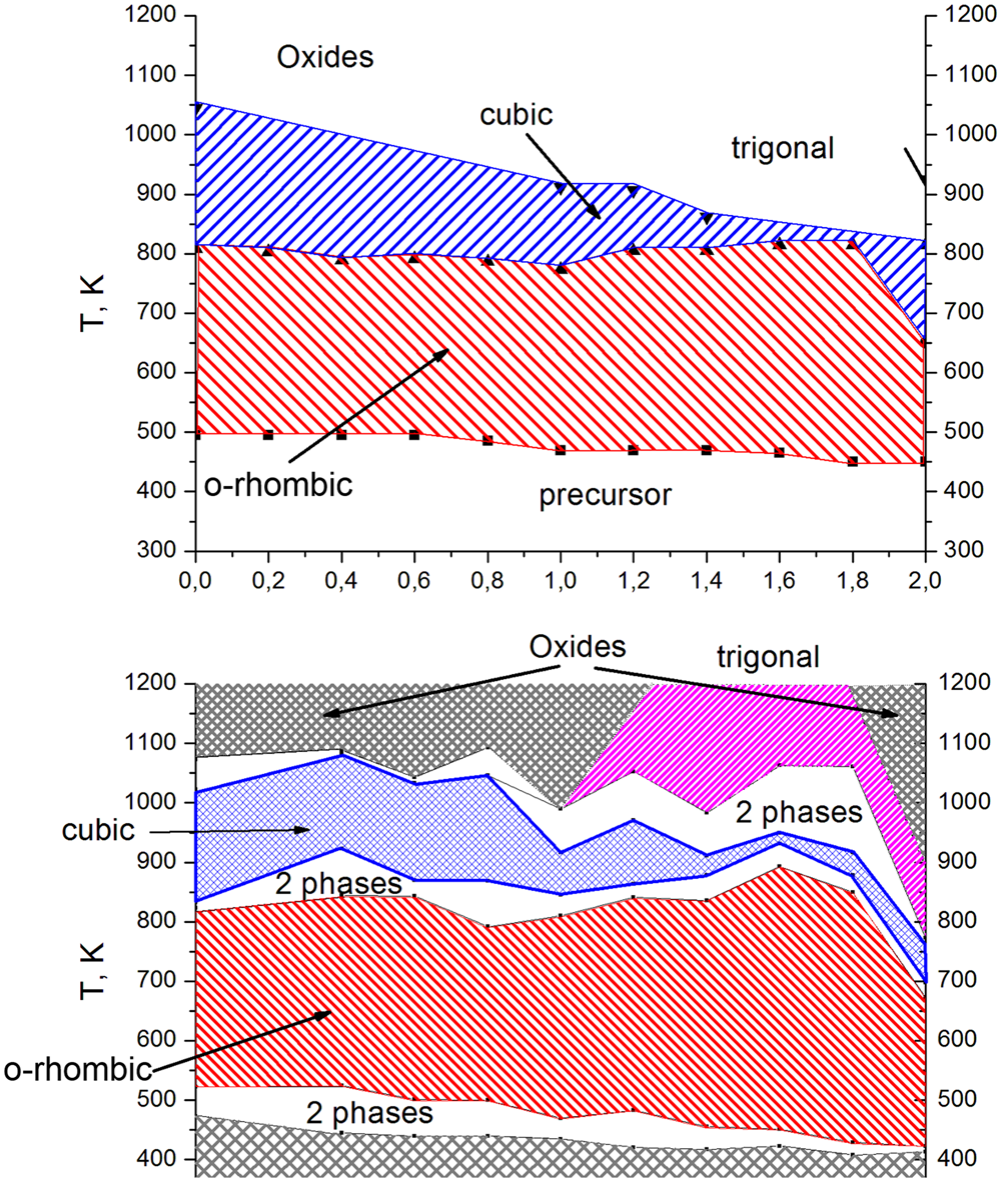
Data on the temperatures of the thermal effect maxima for the thermal degradation of solid solution ZrMo_x_W_2−x_O_7_(OH,Cl)_2_∙2H_2_O obtained by DTA (**a**) and the phases detected by high-temperature PXRD (**b**).

**Figure 3:**
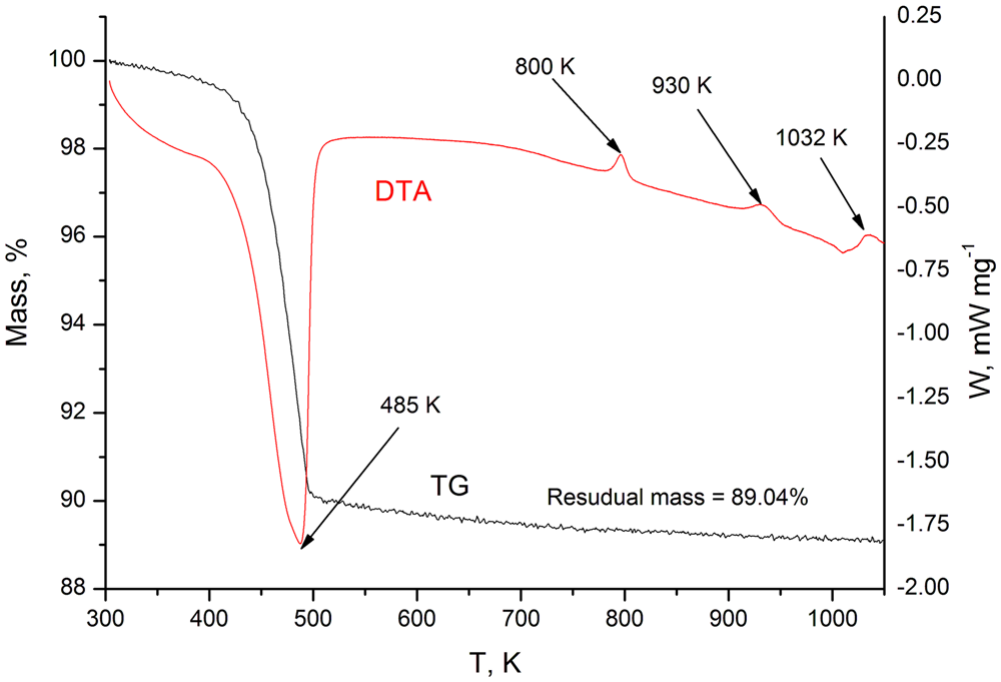
A typical thermogram for ZrMo_x_W_2−x_O_8_ (x = 1.2).

The behaviours of the ZrMo_x_W_2−x_O_7_(OH,Cl)_2_∙2H_2_O samples upon thermal decomposition are similar (Fig. 2a), but some features depend on the value of “*x*”. The decomposition of ZrMo_x_W_2−x_O_7_(OH,Cl)_2_∙2H_2_O begins at 450 K for x = 2 and increases linearly up to 500 K for x = 0. The first stage is accompanied by an endothermic effect and a weight loss (Fig. 3). The data from the high-temperature PXRD confirmed the following reaction:

1 $$\begin{array}{c}{{\rm{Z}}{\rm{r}}{\rm{M}}{\rm{o}}}_{{\rm{x}}}{{\rm{W}}}_{2-{\rm{x}}}{({\rm{O}}{\rm{H}})}_{{\rm{y}}}{{\rm{C}}{\rm{l}}}_{2-{\rm{y}}}\cdot 2{{\rm{H}}}_{2}{\rm{O}}={\rm{o}}{\rm{r}}{\rm{t}}{\rm{h}}{\rm{o}}{\rm{r}}{\rm{h}}{\rm{o}}{\rm{m}}{\rm{b}}{\rm{i}}{\rm{c}}{ \mbox{-} \text{ZrMo}}_{{\rm{x}}}{{\rm{W}}}_{2-{\rm{x}}}{{\rm{O}}}_{8}+(1+{\rm{y}}){{\rm{H}}}_{2}{\rm{O}}+(2-{\rm{y}}){\rm{H}}{\rm{C}}{\rm{l}}\end{array}$$

We calculated the molar ratio of the chloride ion to the hydroxide ion (In all the samples, the ratio was approximately 1:3.) based on the loss of the mass, which was measured by DTA, as described in article^9^.

The diffraction peaks of ZrMo_x_W_2−x_O_7_(OH,Cl)_2_∙2H_2_O decreased to zero, and the diffraction pattern of orthorhombic-ZrMo_x_W_2−x_O_8_ appeared. The peaks of the formed orthorhombic-ZrMo_x_W_2−x_O_8_ had large widths, and the crystallite size was small, approximately 10–15 nm (The Scherer equation was used to calculate the crystallite sizes).

The crystallite sizes did not increase with the temperature in our experiments. Allen^20^ used a long heat treatment (8 hours at 573 K) to prepare good crystalline samples of orthorhombic-ZrMo_x_W_2−x_O_8_.

The second transformation did not have a weight loss and was accompanied by an exothermal effect. The temperatures of the transformation (650–800 K) had a complex dependence on the parameter “*x*” (Fig. 2a). We observed the appearance of the peaks of a nicely crystalline cubic-phase ZrMo_x_W_2−x_O_8_ in the powder pattern. The width of the synthetic “window” for single-phase, cubic ZrMo_x_W_2−x_O_8_ was not large: 70–200 K (x = 0–1.4) and 30–50 K (x = 1.4–2.0). Preparation of pure cubic, single-phase samples required accurate work. The precursor had to be heated to the minimal possible temperature (blue line on Fig. 2b) and held for 10–60 minutes (60 minutes for x = 0 and 10 minutes for x = 2).

Further heating led to the formation of trigonal ZrMo_x_W_2−x_O_8_ at 500–700 K (x = 1.2–2.0) or decomposition of the material into constituent oxides at 1050 K (x = 0–1.0) (Fig. 2b). In study^14^, it was found that the resulting products with x ≤ 0.5 would decompose to WO_3_/MoO_3_ and ZrO_2_ upon a temperature increase, while for x > 0.5, the cubic phase would transform into the trigonal phase. Shi Yongfang^11^ noted that the appearance of the trigonal phase occurs at relatively low temperatures, i.e., 861 K (x = 0.73) and 889 K (x = 0.53). This fact was confirmed in our work. Trace amounts of the trigonal phase were often present in all the PXRD patterns, while the cubic compound was the main phase. However, we did not observe the exothermal effect for the cubic-to-trigonal transformation for x = 0.2–0.8, 1.6, and 1.8 in the DTA experiments.

Trigonal ZrMo_x_W_2−x_O_8_ (x = 1.2–1.8) had a good thermal stability, and we found no evidence of its decomposition for x = 1.4, 1.6 and 1.8 under the conditions in our experiment. Trigonal ZrMo_x_W_2−x_O_8_ (x = 1.2) decomposed at 1150 K. However, trigonal ZrMo_2_O_8_ decayed into oxides with an exo-effect at 925 K.

**Figure 4:**
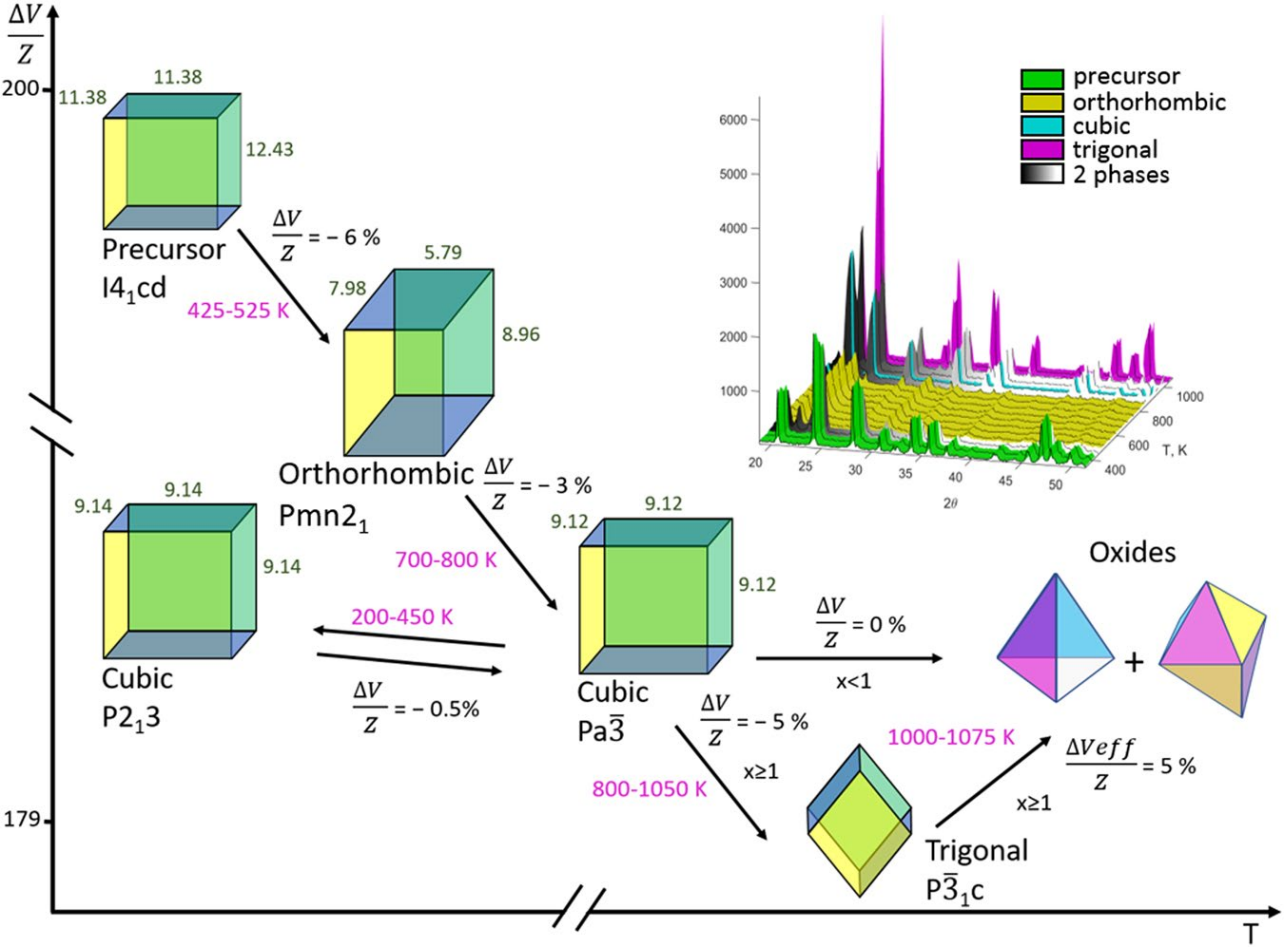
Conversions for the ZrMo_x_W_2−x_O_7_(OH,Cl)_2_∙2H_2_O and ZrMo_x_W_2−x_O_8_ compounds. The inset presents typical data of a high-temperature PXRD; in this case, x = 1.2.

We should note that all the transformations of orthorhombic-cubic-trigonal-oxides are exothermic. This can be explained if all those phases (with the exception of the oxides) are metastable in our experimental temperature ranges. The transformations were accompanied by a decrease in the volume of the formula unit (V/Z), e.g., for x = 1.2 (Fig. 4):

2 $${{\rm{Z}}{\rm{r}}{\rm{M}}{\rm{o}}}_{{\rm{x}}}{{\rm{W}}}_{2-{\rm{x}}}{{\rm{O}}}_{7}{(\text{OH},\text{Cl})}_{2}\cdot 2{{\rm{H}}}_{2}{\rm{O}}\to {\rm{o}}{\rm{r}}{\rm{t}}{\rm{h}}{\rm{o}}{\rm{r}}{\rm{h}}{\rm{o}}{\rm{m}}{\rm{b}}{\rm{i}}{\rm{c}} \mbox{-} {{\rm{Z}}{\rm{r}}{\rm{M}}{\rm{o}}}_{{\rm{x}}}{{\rm{W}}}_{2-{\rm{x}}}{{\rm{O}}}_{8}-6{\rm{ \% }}$$

3 $${\rm{o}}{\rm{r}}{\rm{t}}{\rm{h}}{\rm{o}}{\rm{r}}{\rm{h}}{\rm{o}}{\rm{m}}{\rm{b}}{\rm{i}}{\rm{c}} \mbox{-} {{\rm{Z}}{\rm{r}}{\rm{M}}{\rm{o}}}_{{\rm{x}}}{{\rm{W}}}_{2-{\rm{x}}}{{\rm{O}}}_{8}\cdot {\rm{c}}{\rm{u}}{\rm{b}}{\rm{i}}{\rm{c}} \mbox{-} {{\rm{Z}}{\rm{r}}{\rm{M}}{\rm{o}}}_{{\rm{x}}}{{\rm{W}}}_{2-{\rm{x}}}{{\rm{O}}}_{8}-3{\rm{ \% }}$$

4 $${\rm{c}}{\rm{u}}{\rm{b}}{\rm{i}}{\rm{c}} \mbox{-} {{\rm{Z}}{\rm{r}}{\rm{M}}{\rm{o}}}_{{\rm{x}}}{{\rm{W}}}_{2-{\rm{x}}}{{\rm{O}}}_{8}\cdot {\rm{t}}{\rm{r}}{\rm{i}}{\rm{g}}{\rm{o}}{\rm{n}}{\rm{a}}{\rm{l}} \mbox{-} {{\rm{Z}}{\rm{r}}{\rm{M}}{\rm{o}}}_{{\rm{x}}}{{\rm{W}}}_{2-{\rm{x}}}{{\rm{O}}}_{8}-5{\rm{ \% }}$$

Our data directly indicated the formation of a poorly crystalline orthorhombic phase (Pmn2_1_) of zirconium tungstate (ZrW_2_O_8_) in the temperature range 550–825 K (Fig. 2).

### NTE coefficient of cubic-ZrMo_x_W_2−x_O_8_

The thermal expansion of cubic-ZrMo_x_W_2−x_O_8_ was investigated using variable temperature PXRD. Diffraction data were measured using a Bruker D8 Advance diffractometer (CuKα radiation) with a parallel-beam geometry with Göbel Mirrors. *In situ* experiments were carried out using an Anton Paar XRK900 reaction chamber. The patterns were measured in the 2θ range from 10 to 70° with a step of 0.05° and a collection time of 3 s per point. The heating rate was 12 K/min. The acquisition of the X-ray patterns was started when the given temperature was reached. Sample cooling was immediately performed. The profile analysis and structural refinement by the LeBail method were performed using the TOPAS v.4.3 program^21^, and the data from the powder structural database PDF were used as the standards^16^. The lengths of the coherent scattering domain were calculated using LVol-IB values (i.e., volume weighted mean column height based on the integral breadth).

When measuring the NTE coefficient, it is important to realize which form of cubic ZrMo_x_W_2−x_O_8_ is being measured, i.e., ordered or disordered. For ZrW_2_O_8,_ this transition is called α → β. In article^13^, the thermal effects for the transition (ordered → disordered) were measured, and a formula describing the temperature of the α → β transitions for cubic-ZrMo_x_W_2−x_O_8_ was proposed: T (K) = 432–168.45•x. We used it in our work to estimate whether the NTE coefficient belongs to the corresponding ordered or disordered form (Table 2). Of course, the dependence is not linear for x > 1; this is clearly visible when comparing the calculated and experimental values for x = 1.8 and 2.0 (Table 2).

**Table 2:** Data for cubic ZrMo_x_W_2−x_O_8_: temperature of the ordered to disordered transition, calculated NTE coefficients, crystallite size for the disordered phase.

x	Temperature of (α → β) transformations, K	NTE coefficient, *10^6^, K^−1^		D, nm
		Ordered phase	Disordered phase	
0^5^	430	−8.8	−4.8	
0^9^	400	−6.9	−4.5	
0.2	398*	—	−3.5	34(2)
0.3^14^	380*	−6.61	−6.61	
0.4^8^	373–393	−11.8	−7.4	
0.4	364*	—	−3.5	28(2)
0.6	331*	—	−3.5	31(1)
0.8	297*	—	−3.6	58(1)
1.0	263*	—	−4.5	70(2)
1.0^6^	250	−9.0	−5.5	
1.2	230*	—	−4.5	56(1)
1.4	196*	—	−4.0	60(2)
1.6	162*	—	−4.6	75(4)
1.8	470 (129*)	−9.6	−4.0	114(1)
2.0^7^	200 (95*)	−6.9	−5.0	

*Calculated using formula^13^ T (K) = 432–168.45•x.

We were able to obtain reliable data for the NTE coefficients for only the disordered phase, except for x = 1.8. The calculated coefficients have similar values (from −3.5∙10^−6^ K^−1^ to −4.5∙10^−6^ K^−1^) and almost did not differ from those of the “pure components”. Our value of the NTE coefficient, 3.5∙10^−6^ K^−1^, for x = 0.4 did not coincide with the value^8^ obtained earlier, −7.4∙10^−6^ K^−1^. Only for cubic ZrMo_1.8_W_0.2_O_8_ could the calculated NTE coefficient, −9.6∙10^−6^ K^−1^ (300−400 K), be attributed to the ordered cubic ZrMo_x_W_2−x_O_8_W_2−x_O_8_ (space group P2_1_3).

## Conclusion

All the investigated phases of ZrMo_x_W_2−x_O_7_(OH,Cl)_2_∙2H_2_O and ZrMo_x_W_2−x_O_8_ are metastable at T < 1200 K and P(air) = 1 bar. All transformations that occur during heating are irreversible.

The precursors ZrMo_x_W_2−x_O_7_(OH,Cl)_2_∙2H_2_O (average size of individual particles 50 nm) with pre-determined chemical compositions can be obtained by a hydrothermal method.

Heating of the precursors up to T = 525–50•x K affords crystals of orthorhombic ZrMo_x_W_2−x_O_8_ (10–15 nm).

Cubic ZrMo_x_W_2−x_O_8_ solid solutions with a small crystal grain size (30–100 nm) can be obtained by thermal decomposition of the corresponding precursor or orthorhombic ZrMo_x_W_2−x_O_8_ at a temperature up to 850–900 K at x ≤ 1.8 and T = 2200–750•x K at x > 1.8.

Trigonal ZrMo_x_W_2−x_O_8_ (only for x > 1) can be prepared by heating the precursor or cubic ZrMo_x_W_2−x_O_8_ or orthorhombic ZrMo_x_W_2−x_O_8_ up to 850–1050 K, depending on the exact composition.

Solid solutions of cubic ZrMo_x_W_2−x_O_8_ in the ordered form have a higher coefficient of thermal expansion (CTE) in comparison to that of other materials, but they exist in a very limited and relatively low-temperature range, which limits their possible applications.

The disordered form of cubic ZrMo_x_W_2−x_O_8_ solid solutions does not have an advantage over that of the “pure components” in the value of CTE. The use of solid solutions is justified in the case when it is necessary to have a uniform CTE material for a certain temperature range (e.g., 250–800 K for x = 1.0).

## Electronic supplementary material

Supplementary Information
