# Supplementary material for: Preparation of Zr(Mo,W)2O8 with a larger negative thermal expansion by controlling the thermal decomposition of Zr(Mo,W)2(OH,Cl)2∙2H2O
Source: Sci Rep. 2018 Mar 28;8:5337. doi: 10.1038/s41598-018-23529-6 (PMC5871766; doi:10.1038/s41598-018-23529-6)
Supplement: Supplementary file 1 — Supplementary Information [file 41598_2018_23529_MOESM1_ESM.pdf]

Supplementary Information for

Preparation of $Zr(Mo,W)_2O_8$ with a larger negative thermal expansion by controlling the thermal decomposition of $Zr(Mo,W)_2(OH,Cl)_2·2H_2O$

Mariya Yu. Petrushina[1,2], Elena S. Dedova[3,4], Eugeny Yu. Filatov[1,2], Pavel E. Plyusnin[1,2], Sergei V. Korenev[1,2], Sergei N. Kulkov[3,4], Elizaveta A. Derevyannikova[2,5], Marat R. Sharafutdinov[6,7], Alexander I. Gubanov[*,1,2]

[1]Nikolaev Institute of Inorganic Chemistry, Siberian Branch of the Russian Academy of Sciences, Academician Lavrentiev Prospekt 3, 630090 Novosibirsk, Russian Federation

[2]Novosibirsk State University, Pirogova str. 2, 630090 Novosibirsk, Russian Federation

[3]Institute of Strength Physics and Materials Science, Siberian Branch of the Russian Academy of Sciences, pr. Academicheskii 2/4, 634021 Tomsk, Russian Federation

[4]Tomsk Polytechnic University, Lenin Avenue 30, 634050 Tomsk, Russian Federation

[5]Boreskov Institute of Catalysis, Siberian Branch of the Russian Academy of Sciences, Acad. Lavrentiev Prospekt 5, 630090 Novosibirsk, Russian Federation

[6]Institute of Solid State Chemistry and Mechanochemistry of the Siberian Branch of the Russian Academy of Sciences, Kutateladze 18, 630128 Novosibirsk, Russian Federation

[7]Budker Institute of Nuclear Physics of the Siberian Branch of the Russian Academy of Sciences, Acad. Lavrentiev Prospekt 11, 630090 Novosibirsk, Russian Federation

*E-mail: gubanov@niic.nsc.ru.

This PDF file includes:
Supplementary Figures 4
Supplementary Table 1

Supplementary Figure 1 | A comparison of the PXRD diffraction patter (red dots) for ZrMoxW$_{2-x}$O$_7$(OH,Cl)$_2$·2H$_2$O with calculated by Rietveld refinement model (green solid line).

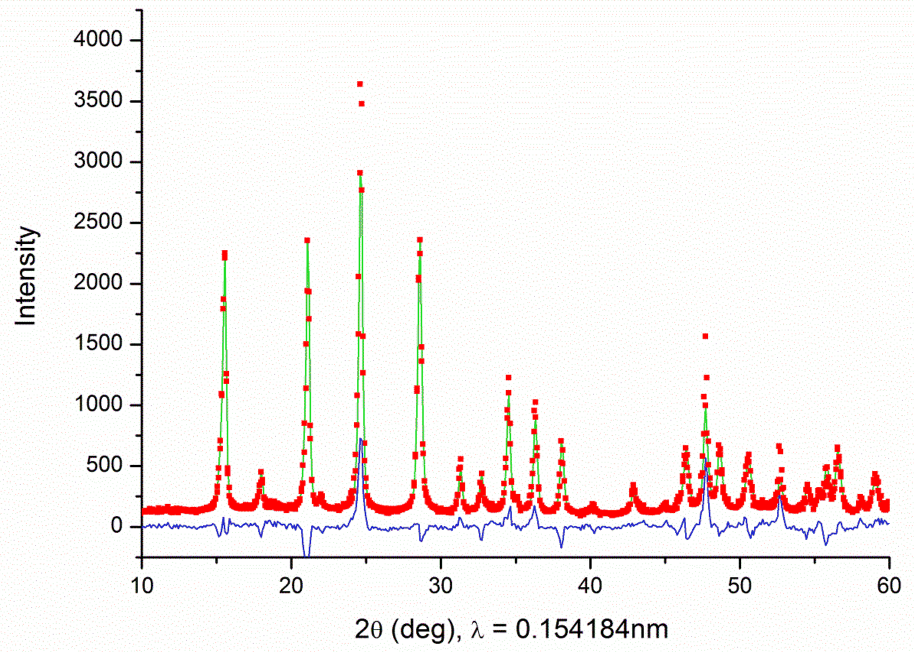

ZrMo$_x$W$_{2-x}$O$_7$(OH,Cl)$_2$·2H$_2$O, x = 0.

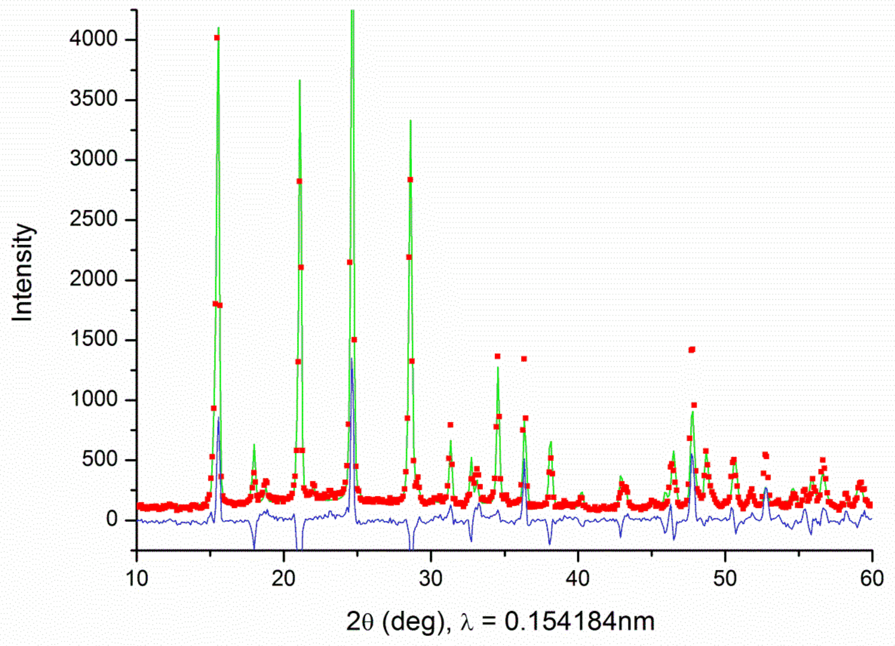

ZrMo$_x$W$_{2-x}$O$_7$(OH,Cl)$_2$·2H$_2$O, x = 0.2.

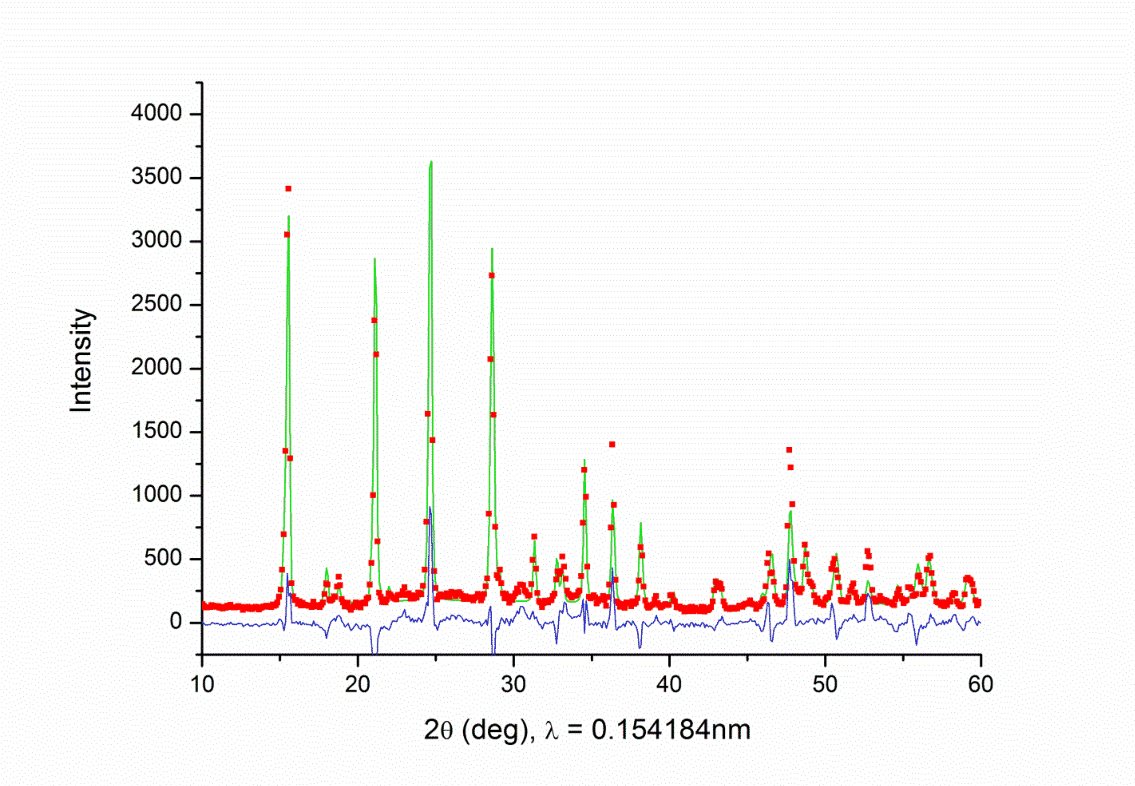

$ZrMo_xW_{2-x}O_7(OH,Cl)_2 \cdot 2H_2O$, x = 0.4.

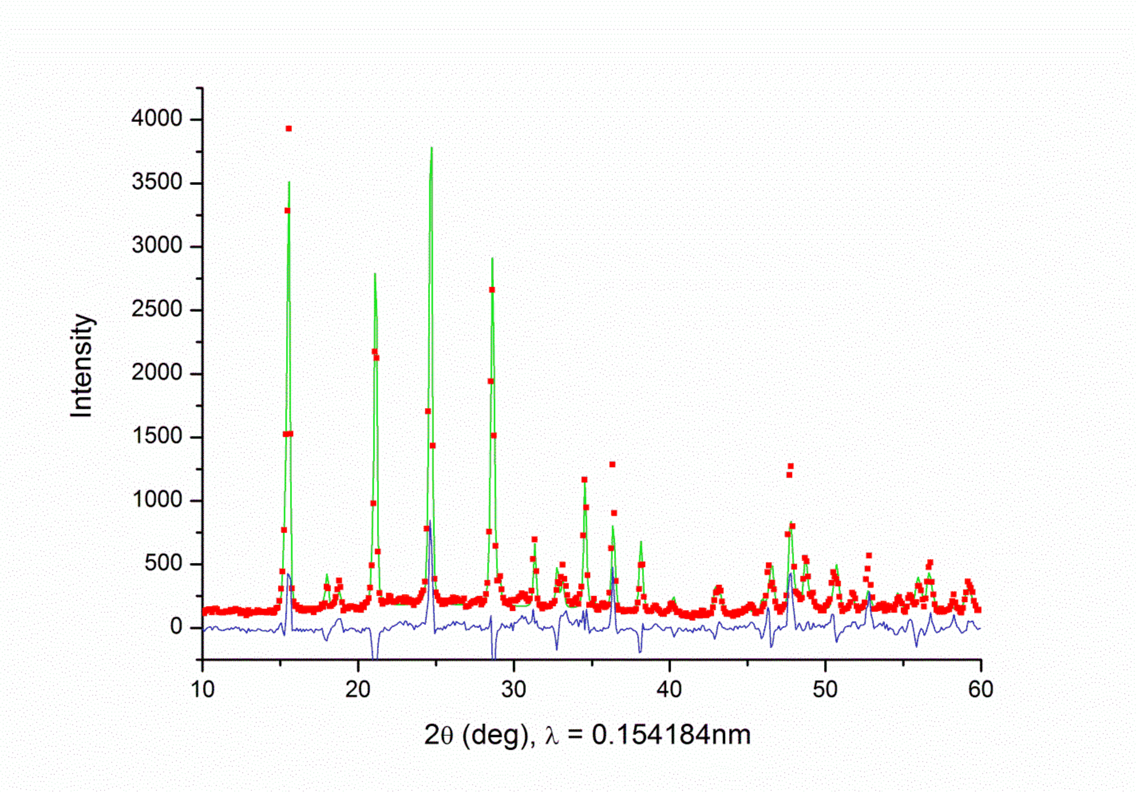

$ZrMo_xW_{2-x}O_7(OH,Cl)_2 \cdot 2H_2O$, x = 0.6.

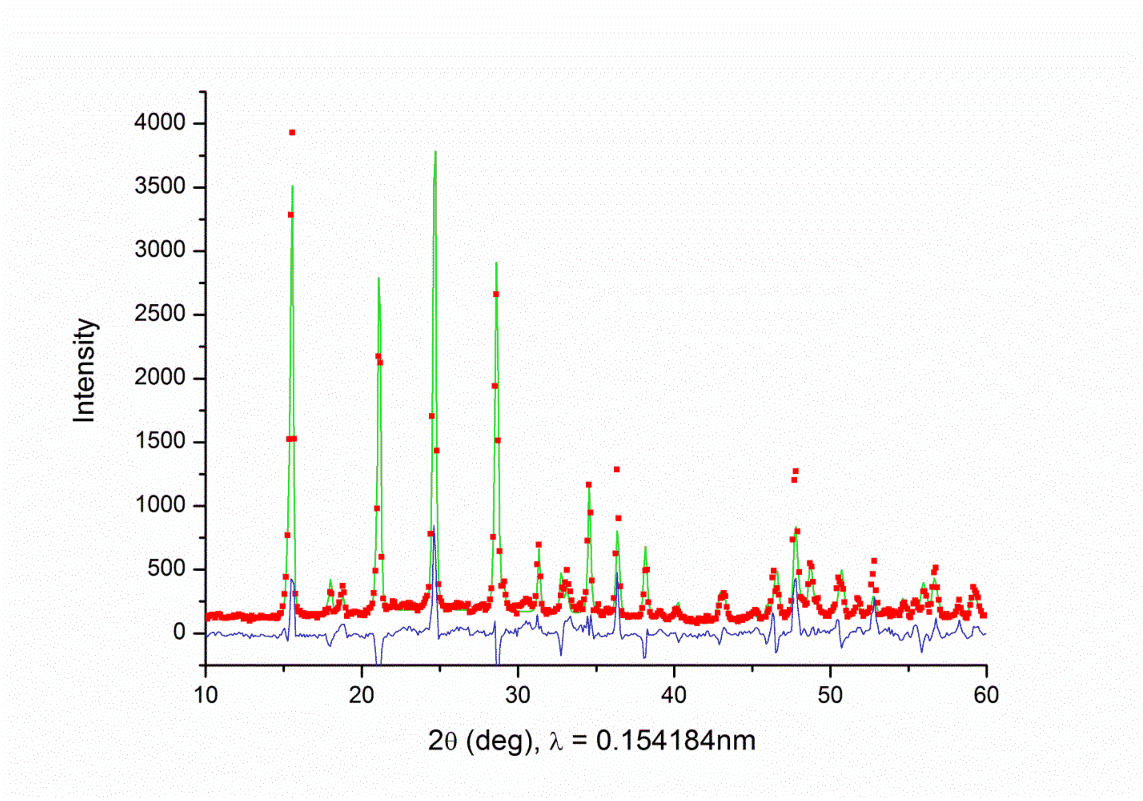

$ZrMo_xW_{2-x}O_7(OH,Cl)_2 \cdot 2H_2O$, x = 0.8.

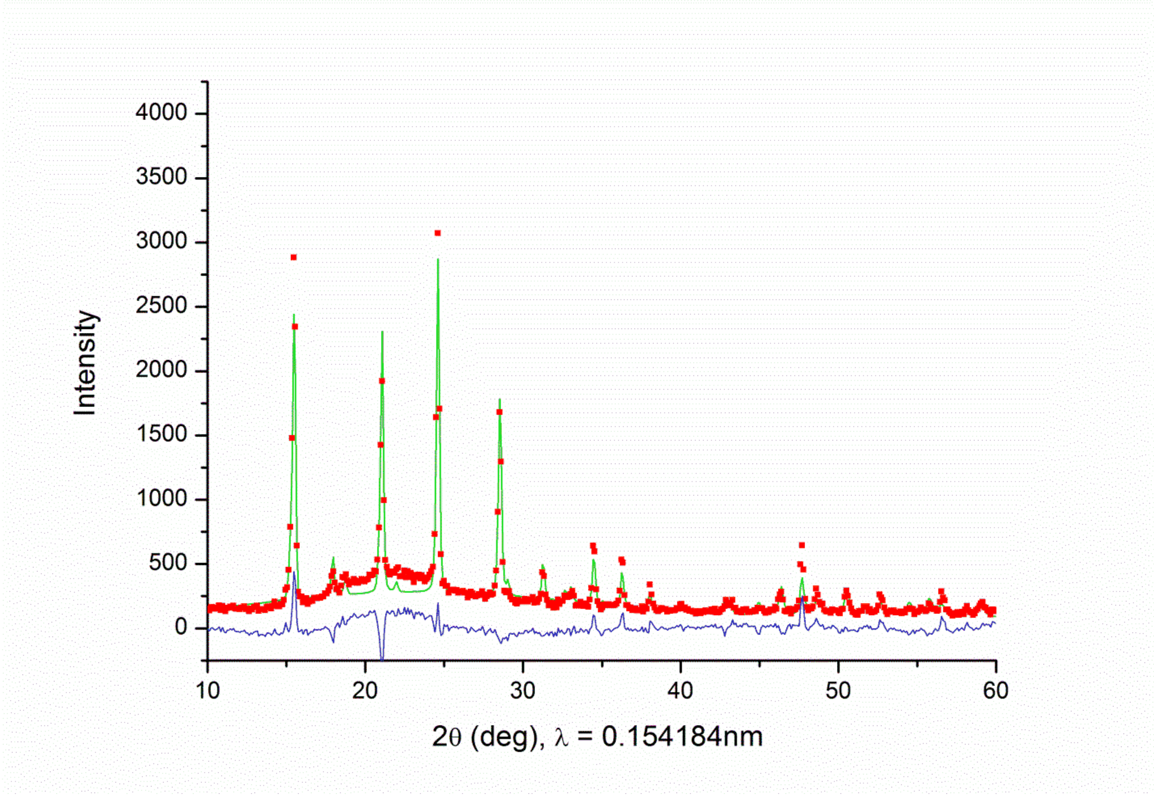

$ZrMo_xW_{2-x}O_7(OH,Cl)_2 \cdot 2H_2O$, x = 1.0.

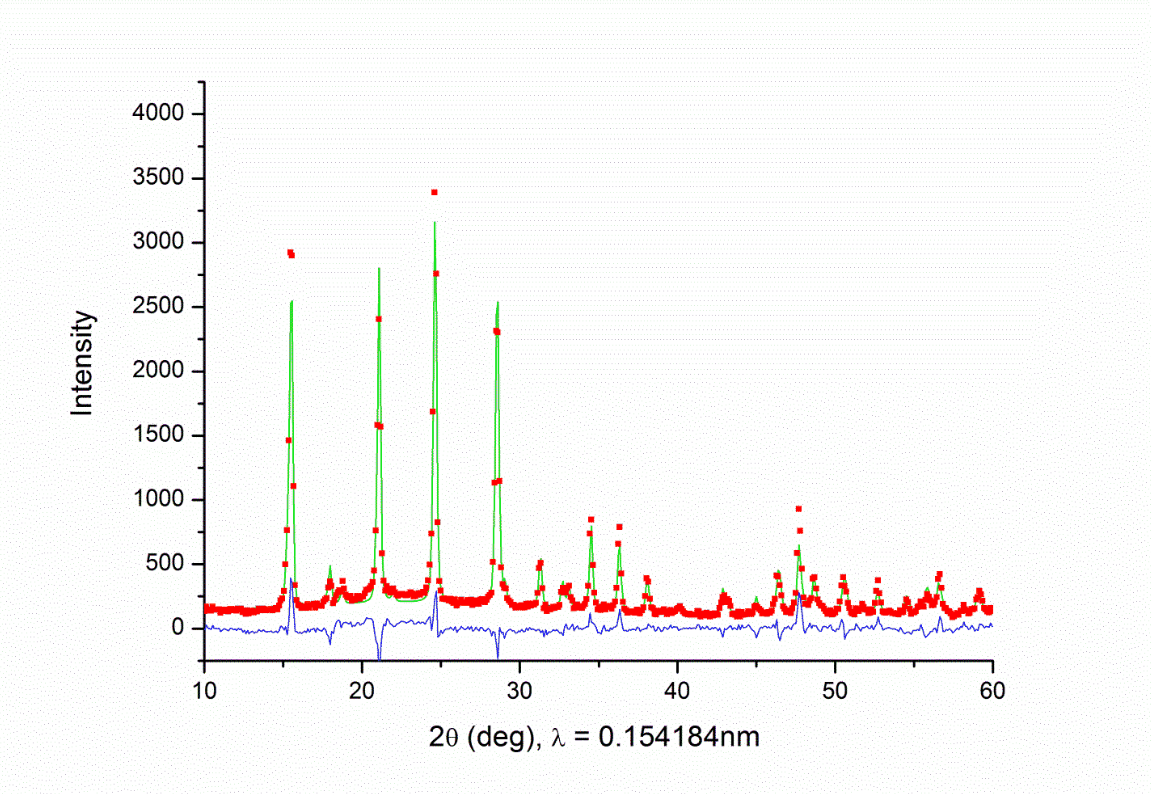

$ZrMo_xW_{2-x}O_7(OH,Cl)_2 \cdot 2H_2O$, x = 1.2.

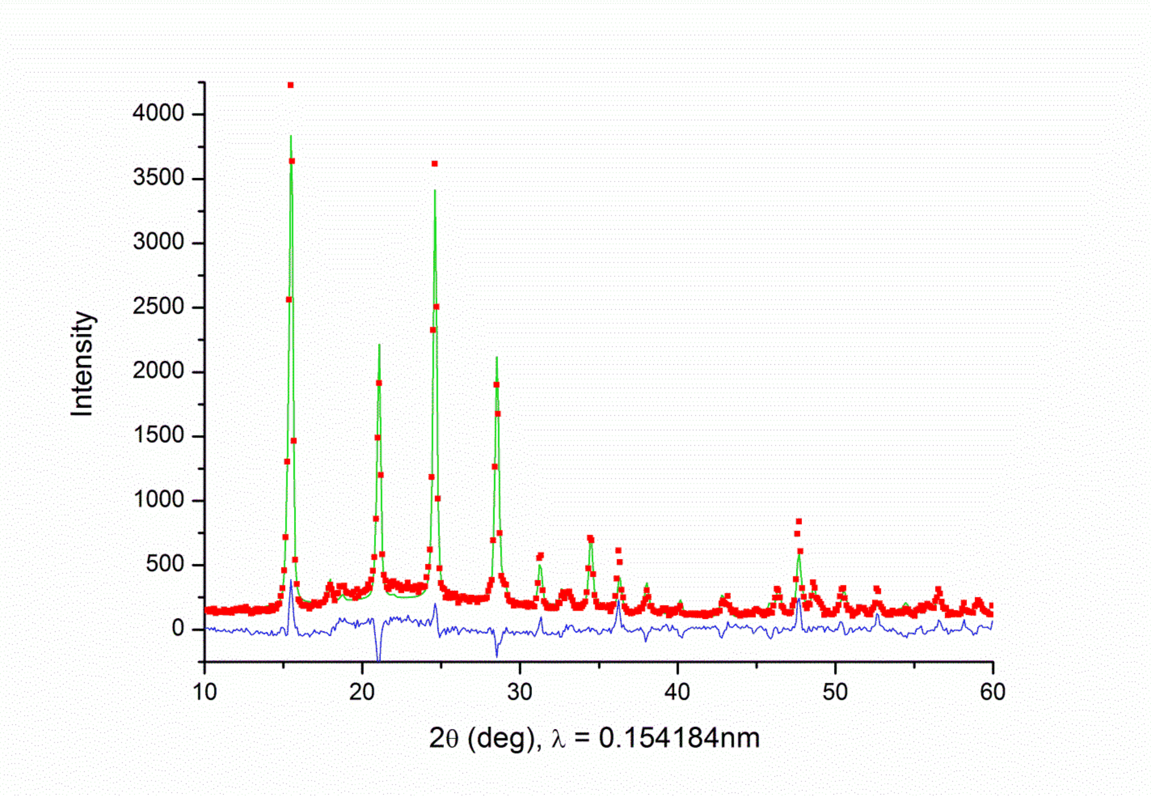

$ZrMo_xW_{2-x}O_7(OH,Cl)_2 \cdot 2H_2O$, x = 1.4.

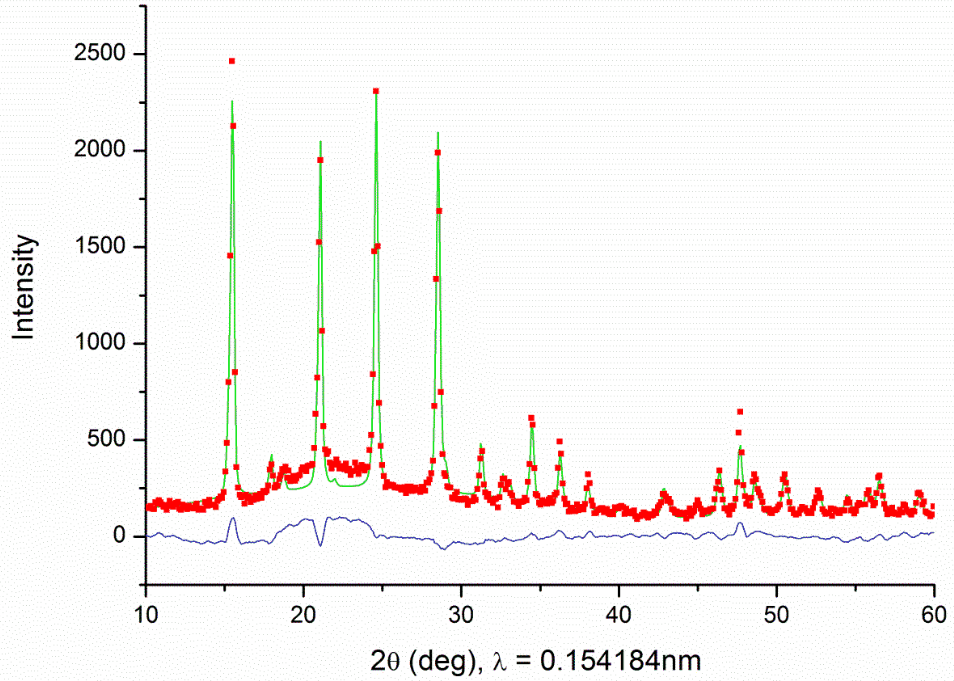

$ZrMo_xW_{2-x}O_7(OH,Cl)_2 \cdot 2H_2O$, x = 1.6.

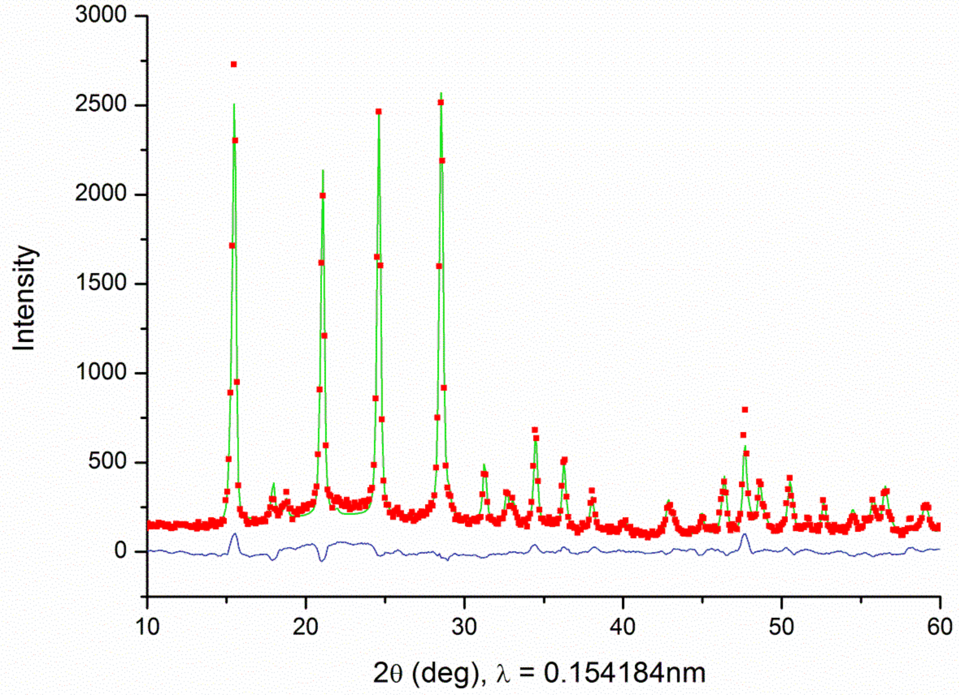

$ZrMo_xW_{2-x}O_7(OH,Cl)_2 \cdot 2H_2O$, x = 1.8.

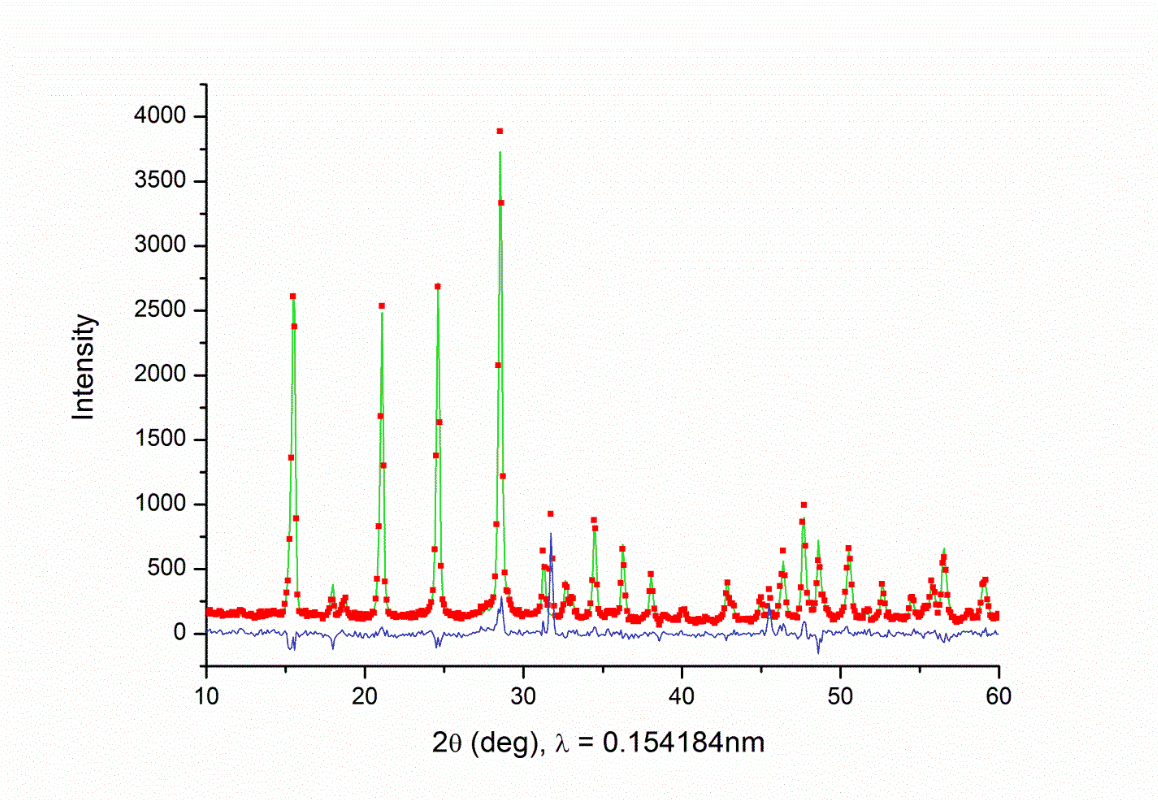

$ZrMo_xW_{2-x}O_7(OH,Cl)_2 \cdot 2H_2O$, x = 2.0.

Supplementary Figure 2 | Thermograms for $ZrMo_xW_{2-x}O_8$Red line – DTA (DSC), Black line – TG and Comparison of the thermograms of $ZrMo_2O_7(OH,Cl)_2{\cdot}2H_2O$ and $ZrW_2O_7(OH,Cl)_2{\cdot}2H_2O$

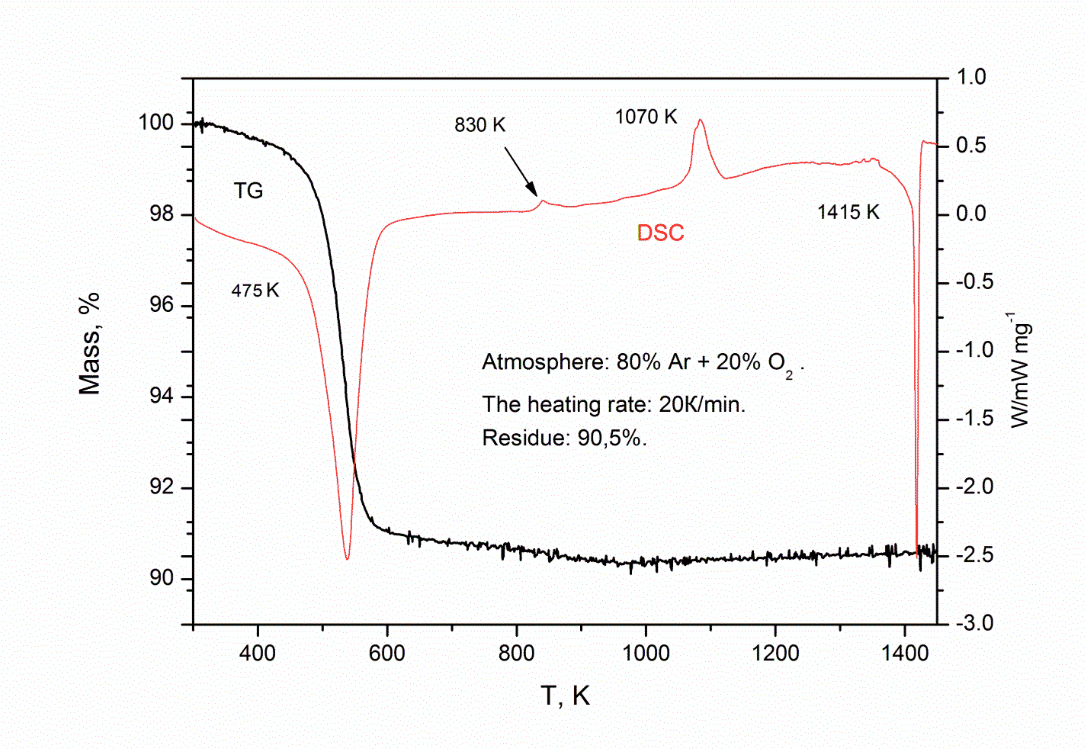

$ZrMo_xW_{2-x}O_7(OH,Cl)_2{\cdot}2H_2O$, x = 0.

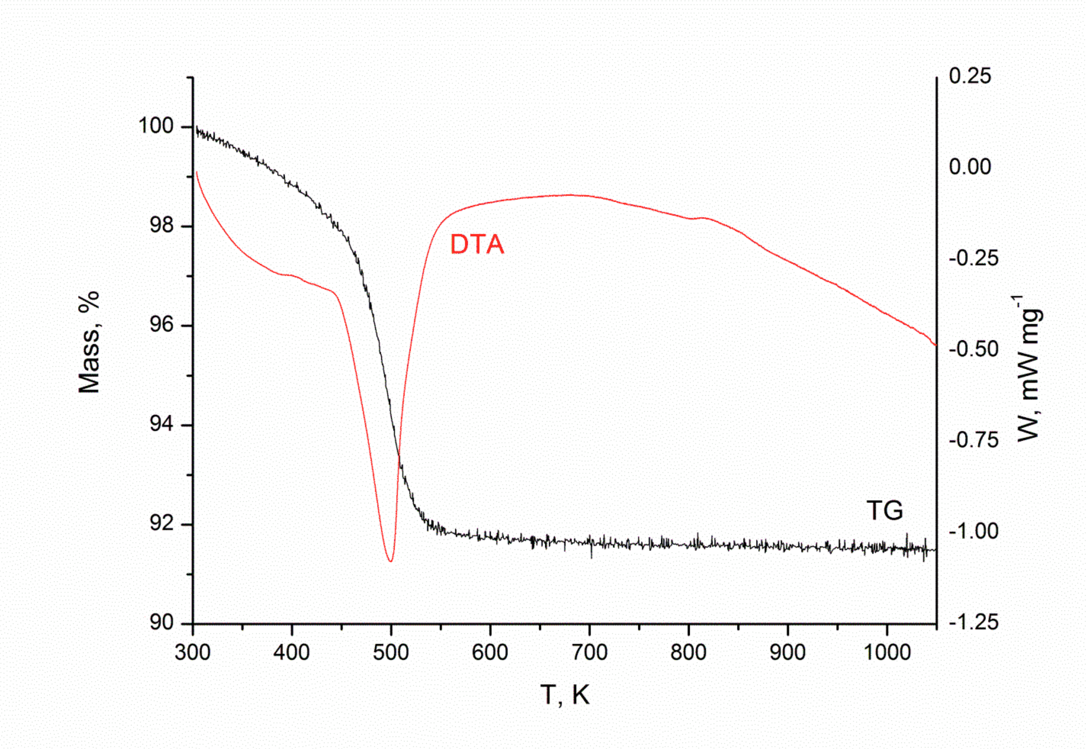

$ZrMo_xW_{2-x}O_7(OH,Cl)_2{\cdot}2H_2O$, x = 0.2.

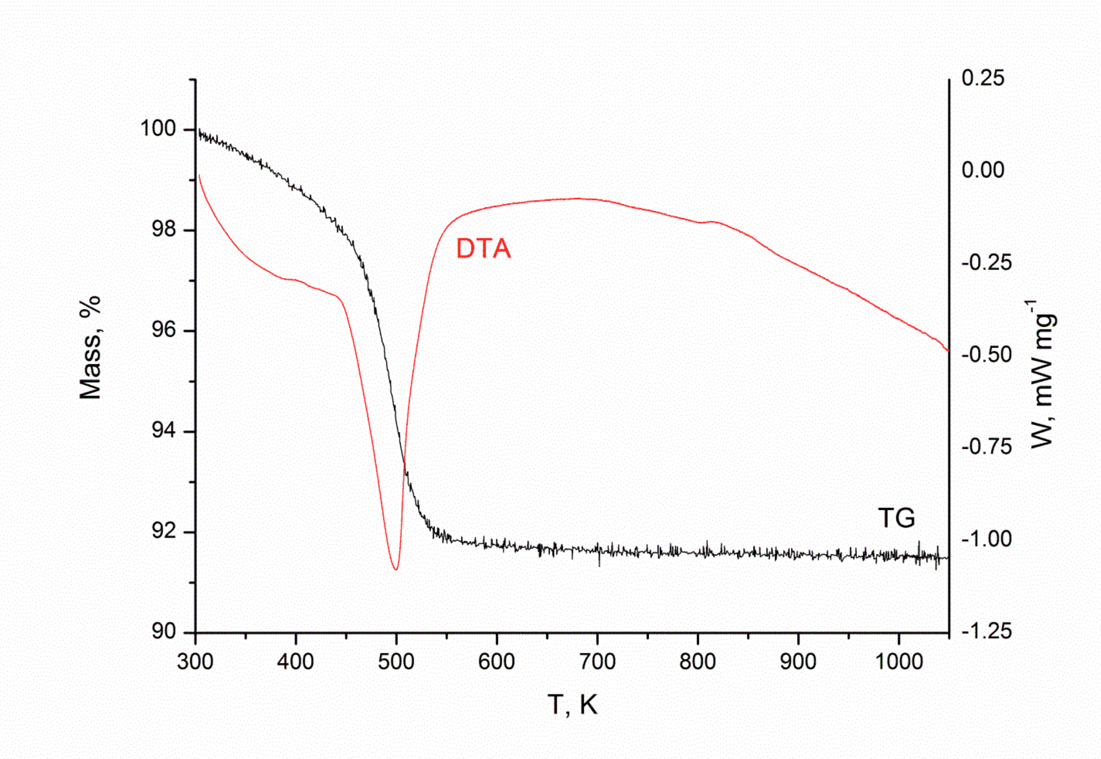

$ZrMo_xW_{2-x}O_7(OH,Cl)_2 \cdot 2H_2O$, x = 0.4.

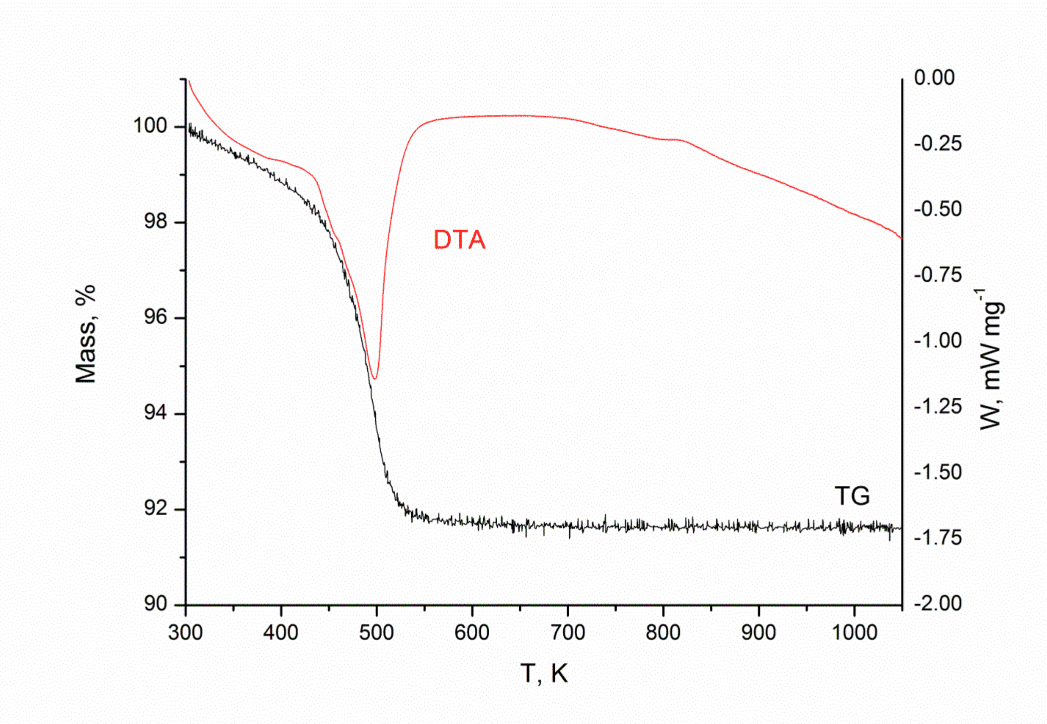

$ZrMo_xW_{2-x}O_7(OH,Cl)_2 \cdot 2H_2O$, x = 0.6.

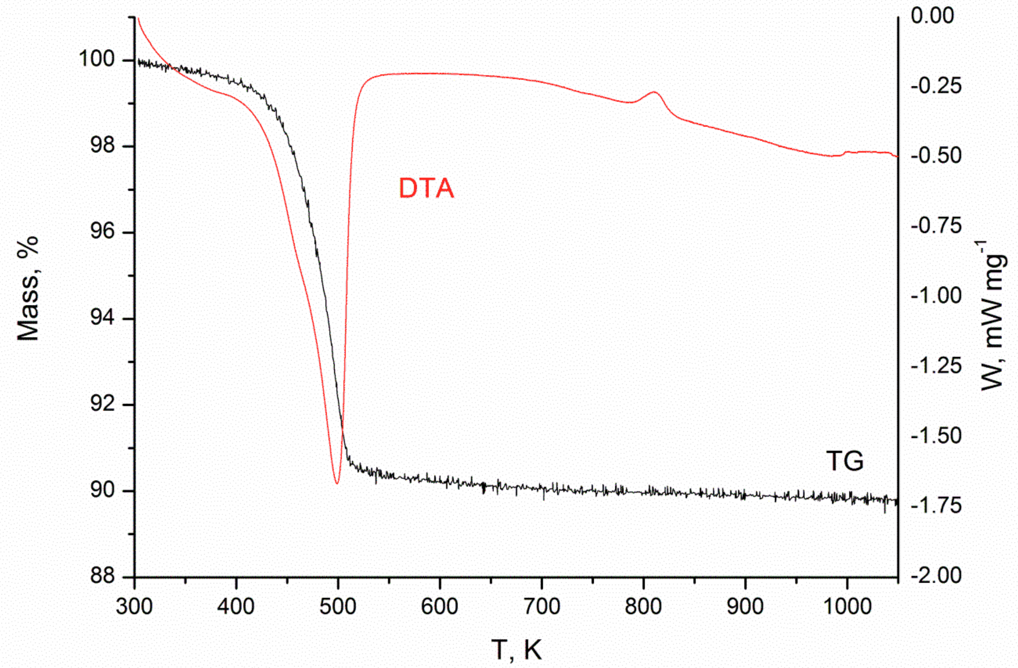

$ZrMo_xW_{2-x}O_7(OH,Cl)_2 \cdot 2H_2O$, x = 0.8.

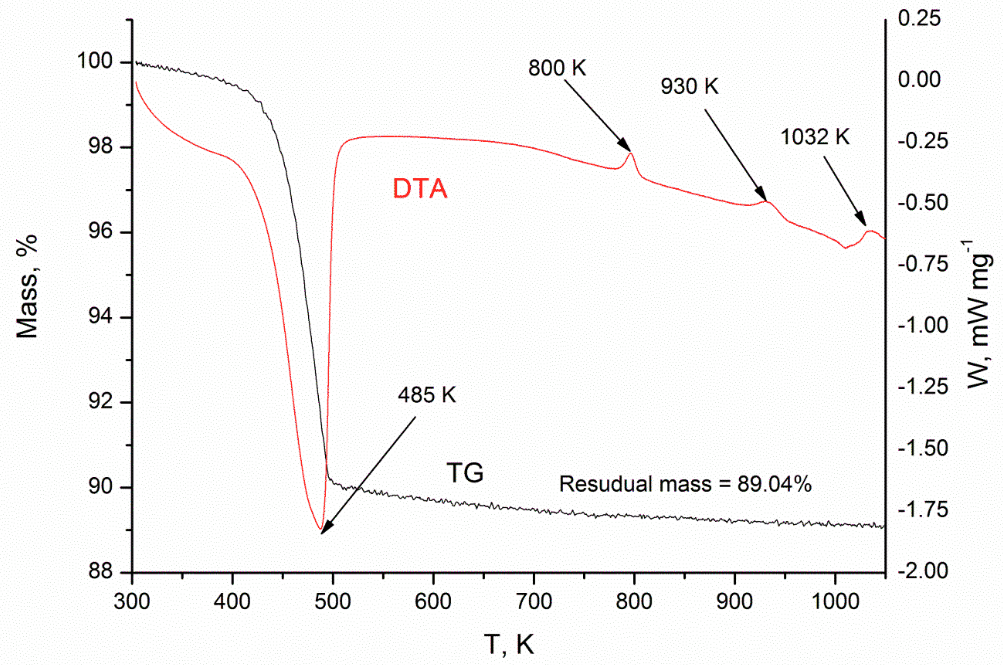

$ZrMo_xW_{2-x}O_7(OH,Cl)_2 \cdot 2H_2O$, x = 1.0.

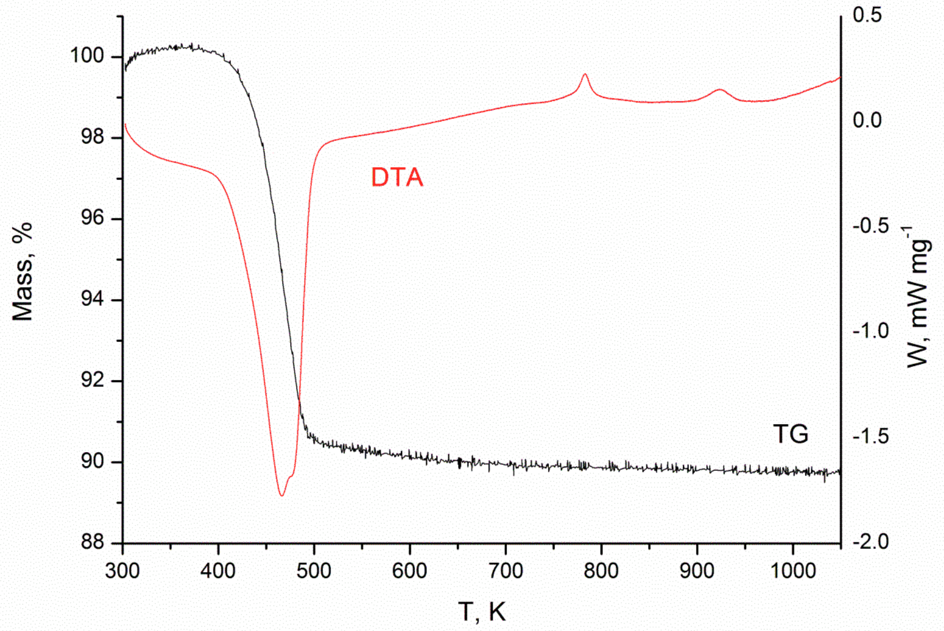

$ZrMo_xW_{2-x}O_7(OH,Cl)_2·2H_2O$, x = 1.2.

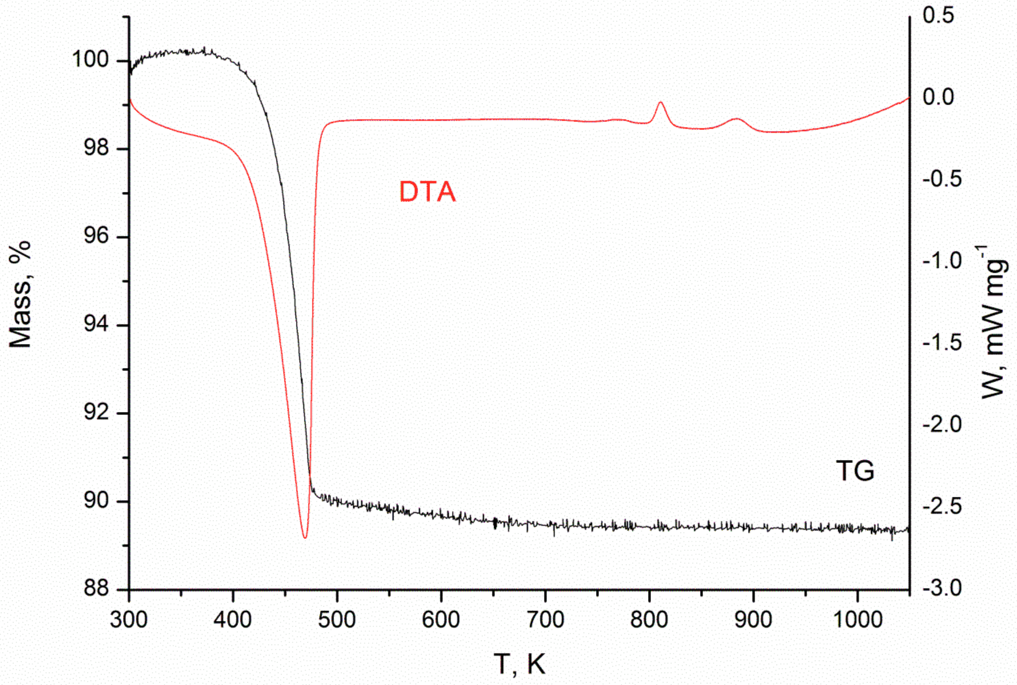

$ZrMo_xW_{2-x}O_7(OH,Cl)_2·2H_2O$, x = 1.4.

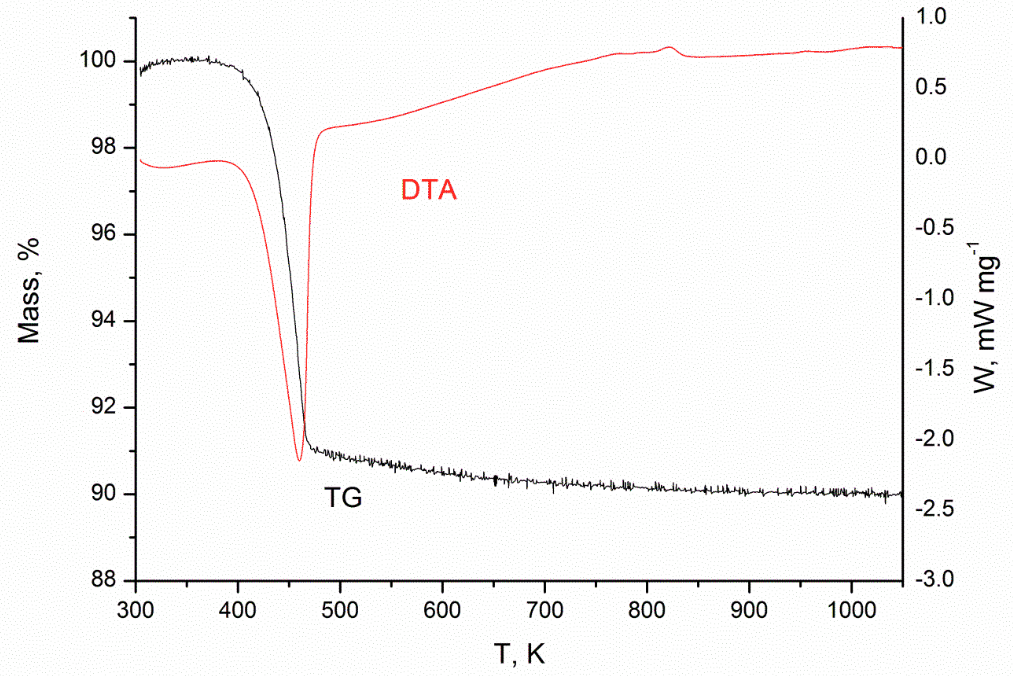

$ZrMo_xW_{2-x}O_7(OH,Cl)_2 \cdot 2H_2O$, x = 1.6.

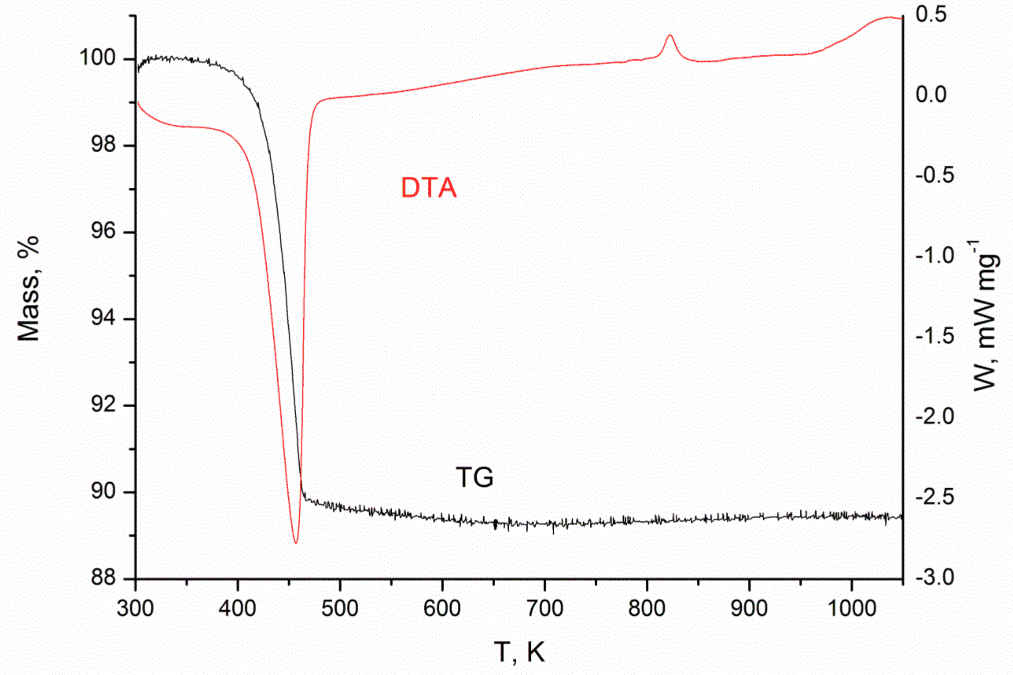

$ZrMo_xW_{2-x}O_7(OH,Cl)_2 \cdot 2H_2O$, x = 1.8

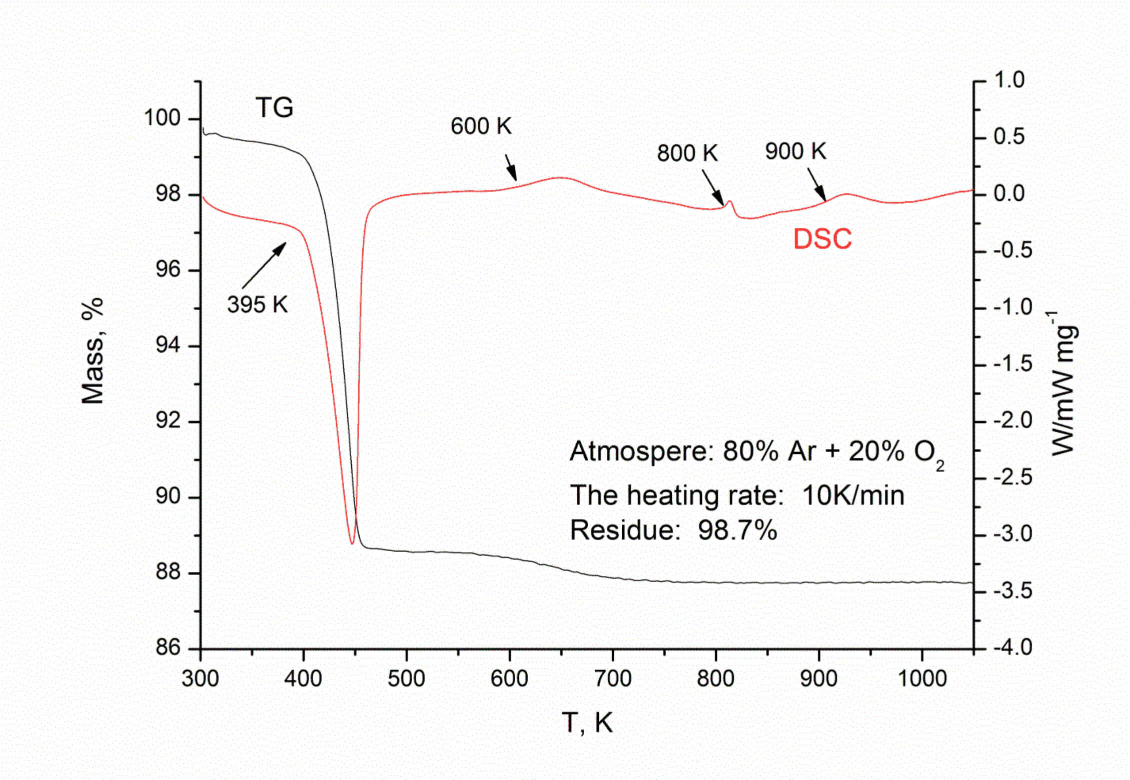

$ZrMo_xW_{2-x}O_7(OH,Cl)_2{\cdot}2H_2O$, x = 2.0

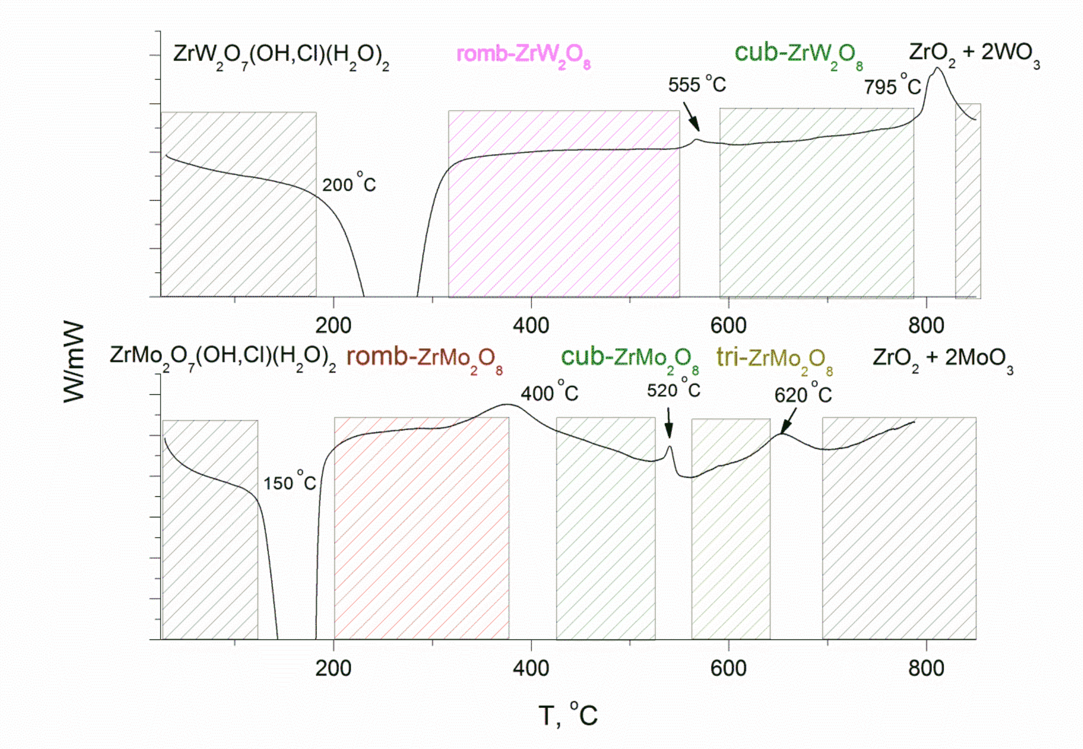

Comparison of the thermograms of $ZrMo_2O_7(OH,Cl)_2{\cdot}2H_2O$ and $ZrW_2O_7(OH,Cl)_2{\cdot}2H_2O$ with the attribution of thermolysis products to certain temperatures. Attention – temperature is given in degrees Celsius.

Supplementary Figure 3 | Data of EDX experiments for ZrMoxW$_{2-x}$O$_7$(OH,Cl)$_2$·2H$_2$O

ZrMo$_x$W$_{2-x}$O7(OH,Cl)$_2$·2H$_2$O, x = 0.2

Point 1.

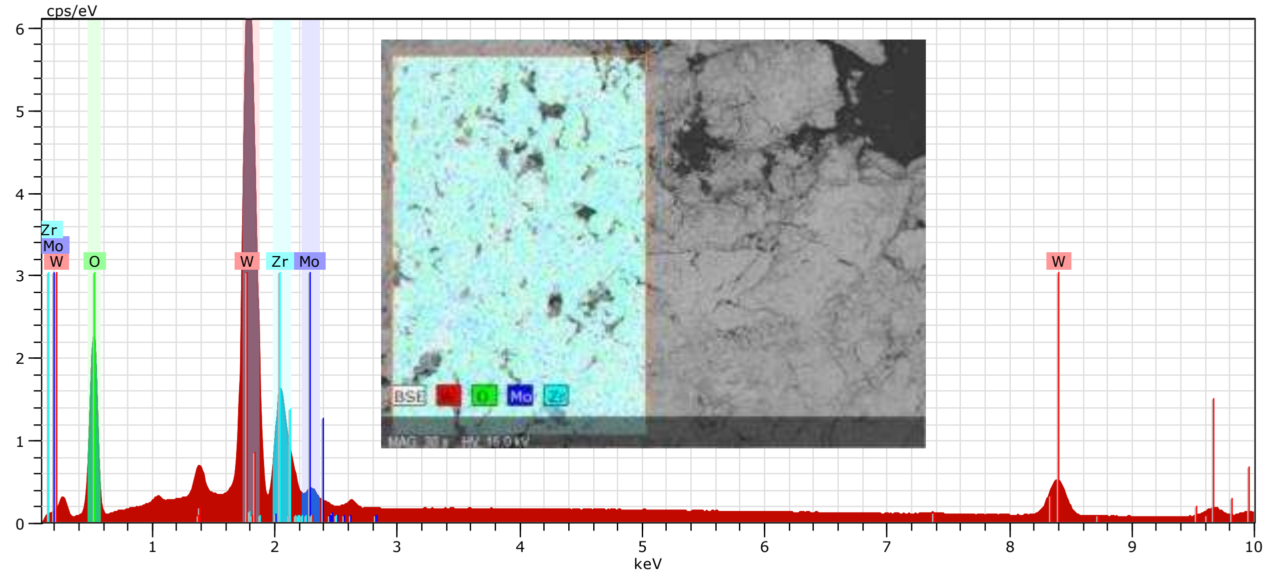

Element	AN	[norm. at.%]	Error in %
Tungsten	74	87,6	2,8
Molybdenum	42	12,4	0,20
		100	

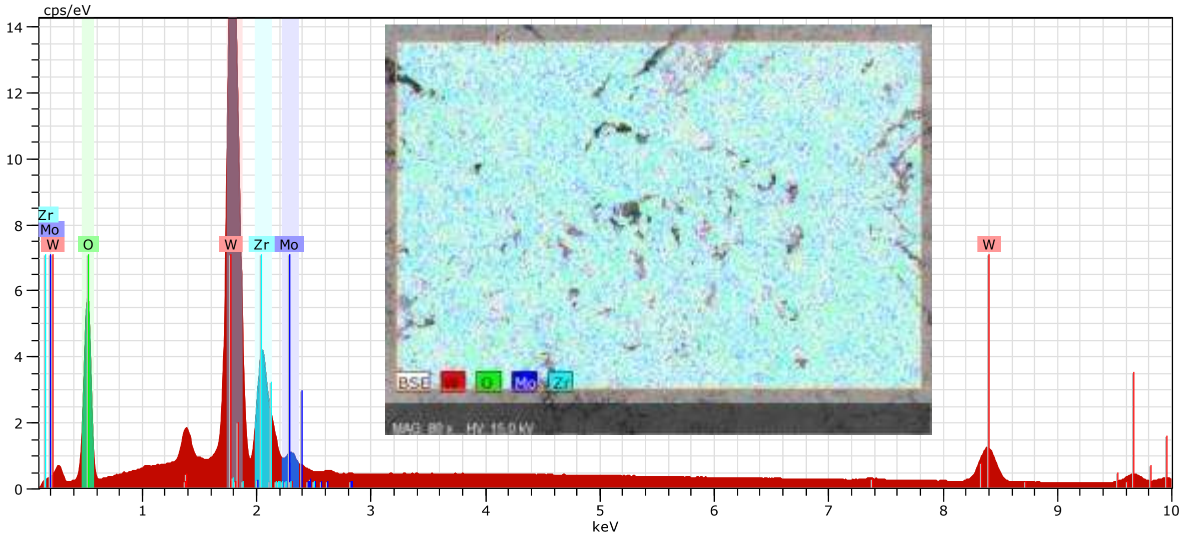

Element	AN	[norm. at.%]	Error in %
Tungsten	74	87,6	2,8
Molybdenum	42	12,4	0,2
		100	

$ZrMo_xW_{2-x}O7(OH,Cl)_2 \cdot 2H_2O$, x = 0.4

Point 1.

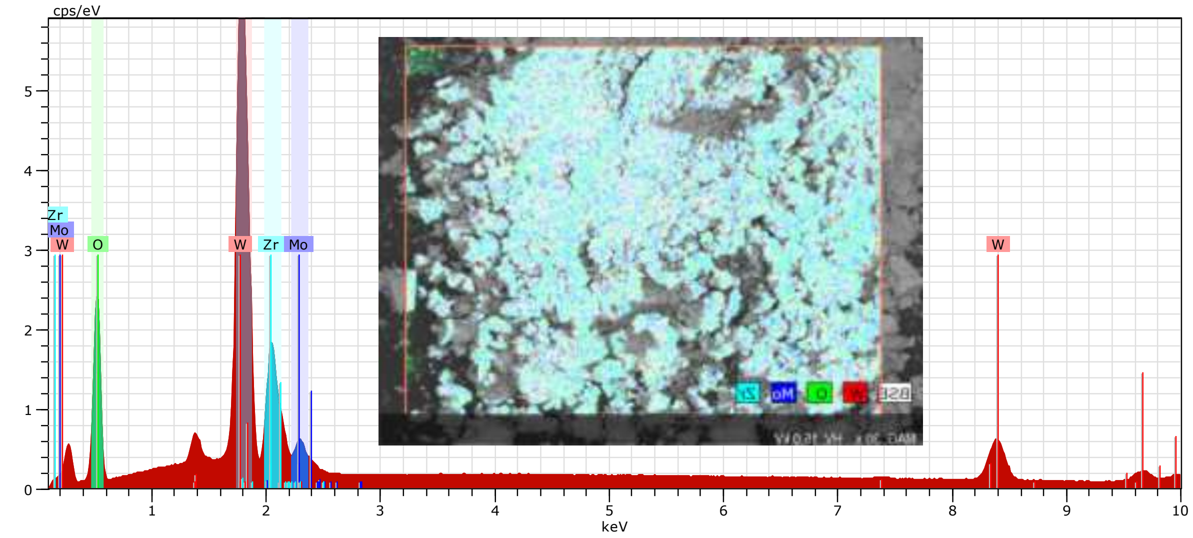

Element	AN	[norm. at.%]	Error in %
Tungsten	74	78,6	2,4
Molybdenum	42	21,4	0,3
		100	

Point 2.

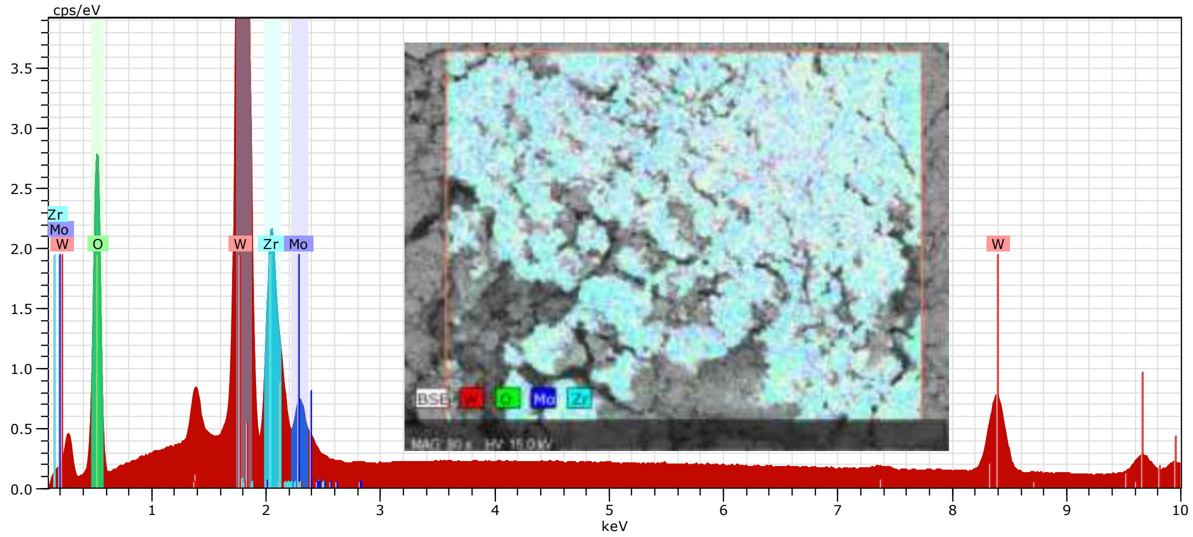

Element	AN	[norm. at.%]	Error in %
Tungsten	74	80,3	2,5
Molybdenum	42	19,7	0,3
		100	

$ZrMo_xW_{2-x}O7(OH,Cl)_2\cdot 2H_2O$, $x = 0.6$

Point 1.

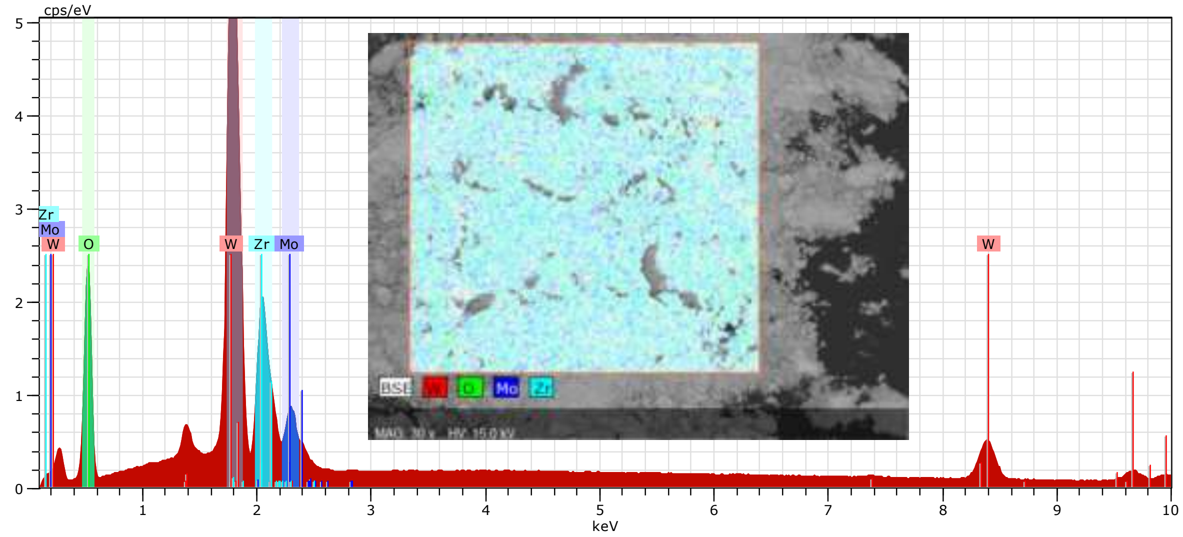

Element	AN	[norm. at.%]	Error in %
Tungsten	74	70,8	2,2
Molybdenum	42	29,2	0,4
		100	

Point 2.

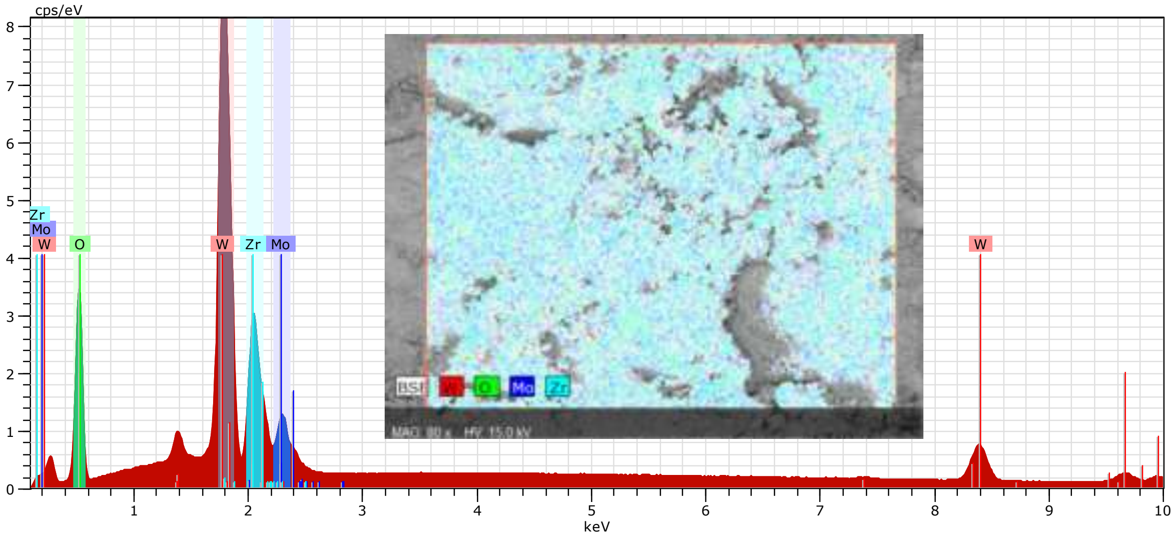

Element	AN	[norm. at.%]	Error in %
Tungsten	74	70,4	2,2
Molybdenum	42	29,6	0,4

		100	

$ZrMo_xW_{2-x}O7(OH,Cl)_2{\cdot}2H_2O$, x = 0.8

Point 1.

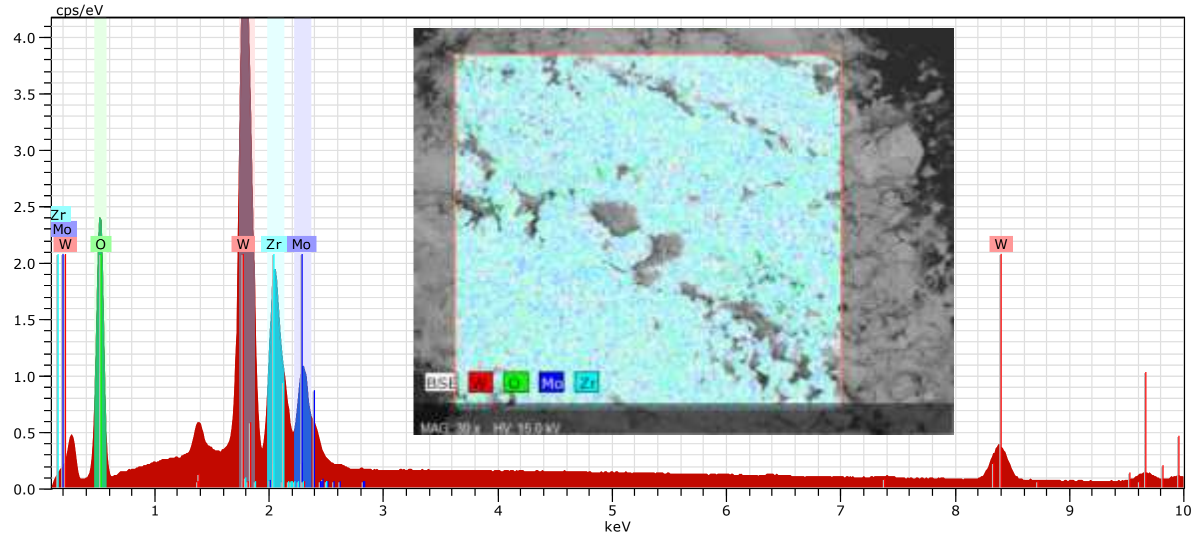

Element	AN	[norm. at.%]	Error in %
Tungsten	74	59,6	1,9
Molybdenum	42	41,4	0,6
		100	

Point 2.

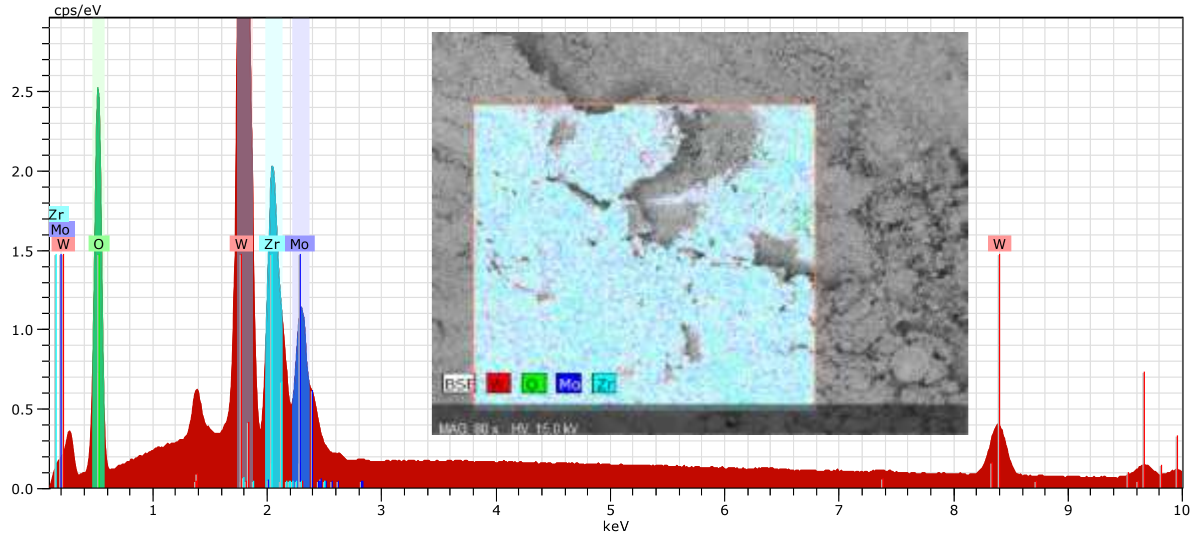

Element	AN	[norm. at.%]	Error in %
Tungsten	74	60, 0	1,9
Molybdenum	42	40,0	0,6
		100	

$ZrMo_xW_{2-x}O7(OH,Cl)_2 \cdot 2H_2O$, x = 1.0

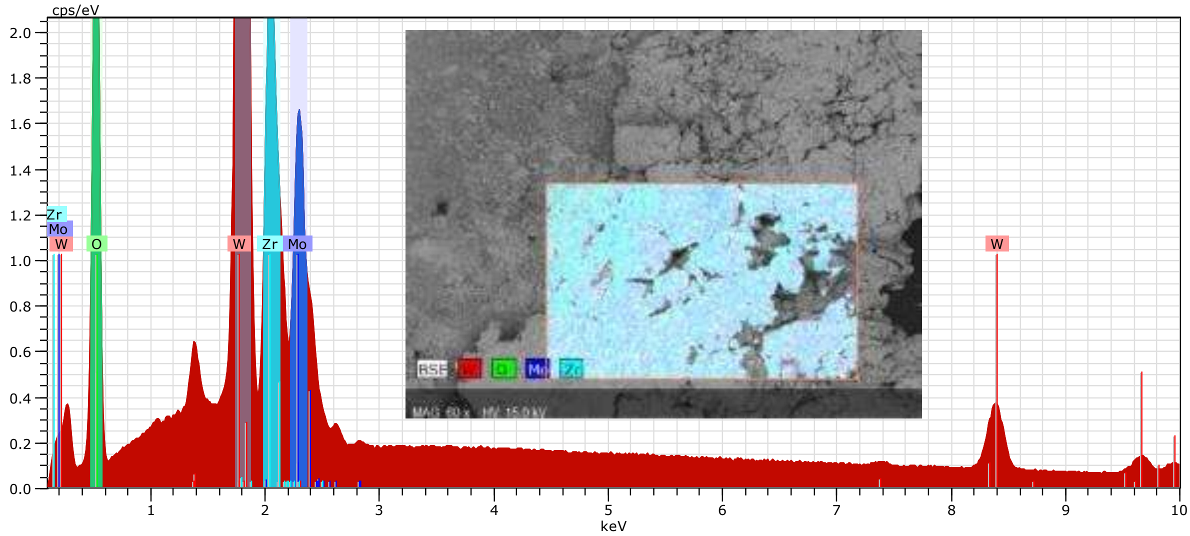

Element	AN	[norm. at.%]	Error in %
Tungsten	74	50,3	1,9
Molybdenum	42	49,7	0,8
		100	

$ZrMo_xW_{2-x}O7(OH,Cl)_2 \cdot 2H_2O$, x = 1.2

Point 1.

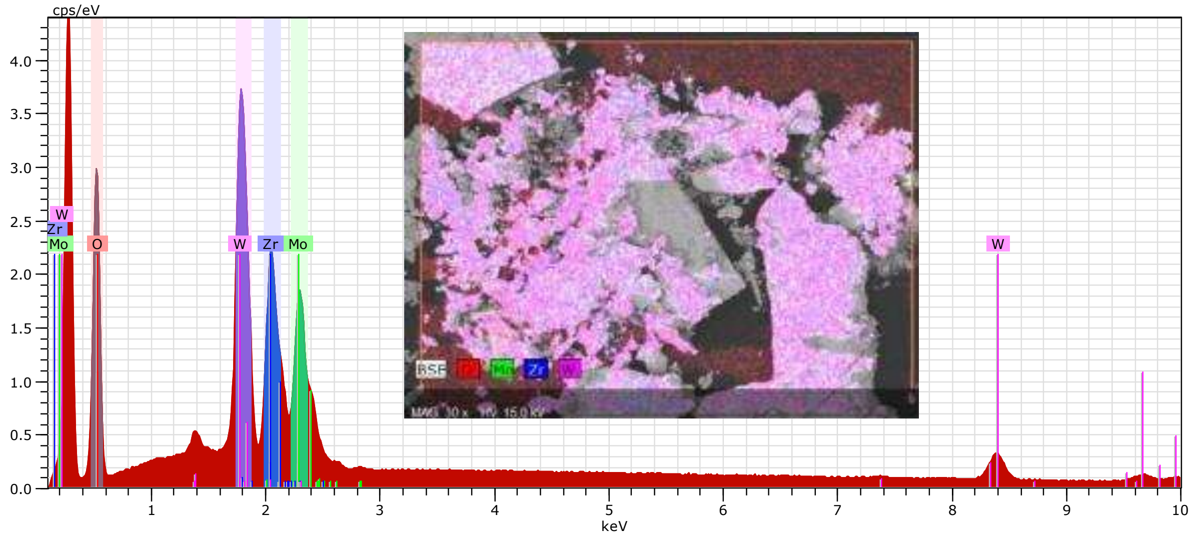

Element	AN	[norm. at.%]	Error in %
Tungsten	74	39,4	1,2
Molybdenum	42	60,6	0,8
		100	

Point 2.

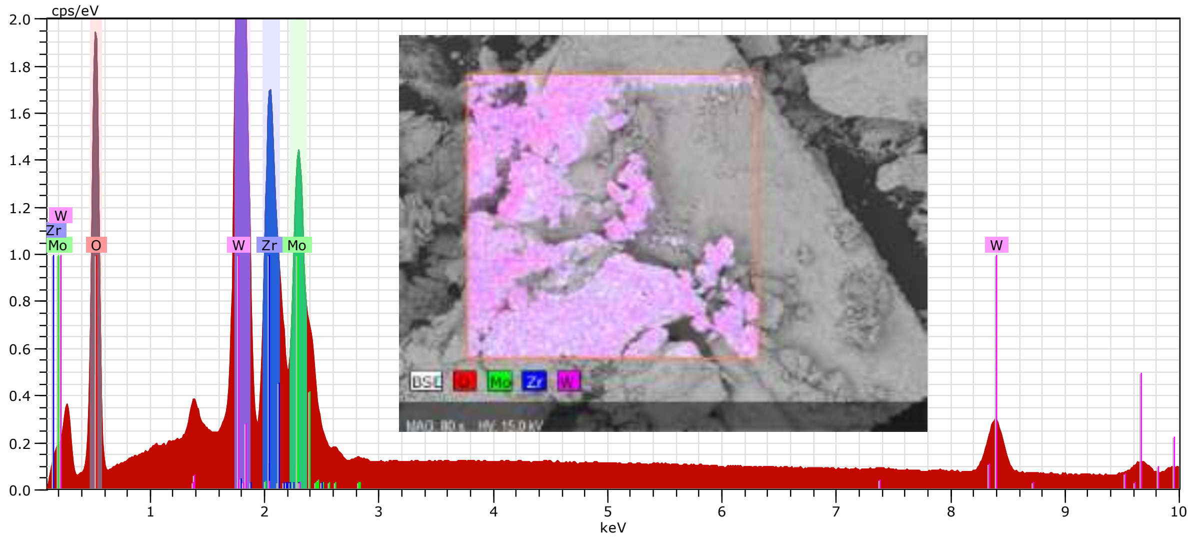

Element	AN	[norm. at.%]	Error in %
Tungsten	74	40,0	1,5
Molybdenum	42	60,0	0,9
		100	

$ZrMo_xW_{2-x}O7(OH,Cl)_2 \cdot 2H_2O$, x = 1.4

Point 1.

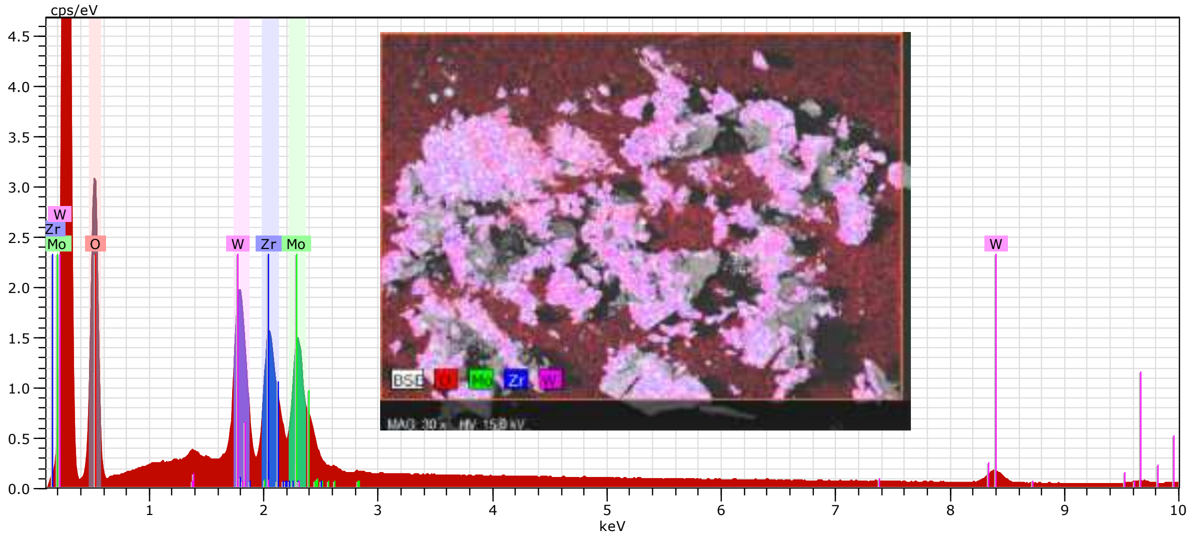

Element	AN	[norm. at.%]	Error in %
Molybdenum	42	70, 0	0,7
Tungsten	74	30,0	0,6
		100	

Point 2.

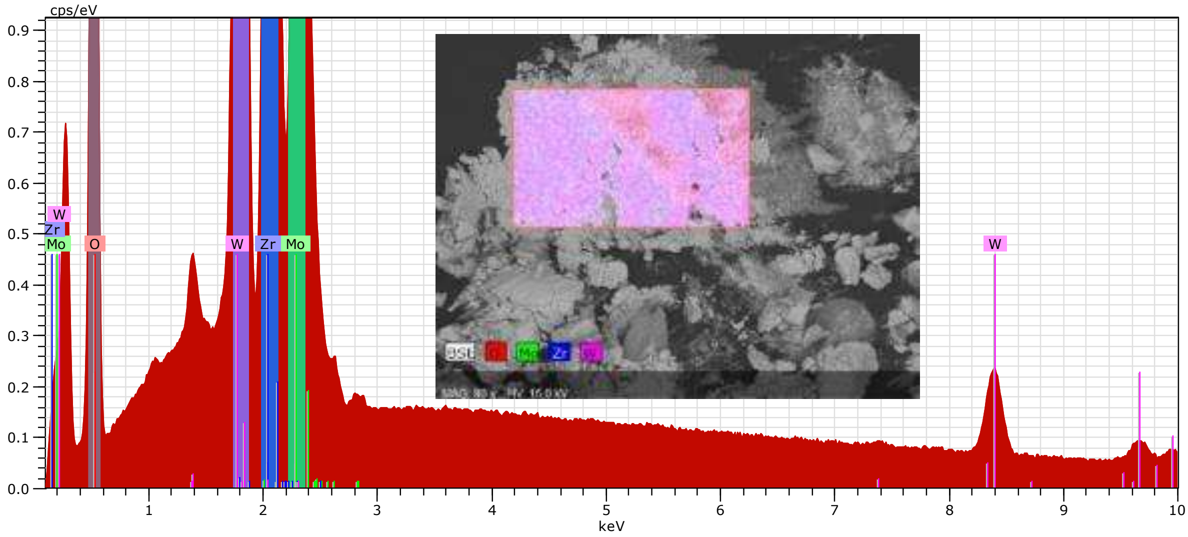

Element	AN	[norm. at.%]	Error in %
Molybdenum	42	68,9	1,1
Tungsten	74	31,1	1,1
		100	

$ZrMo_xW_{2-x}O7(OH,Cl)_2 \cdot 2H_2O$, x = 1.6

Point 1.

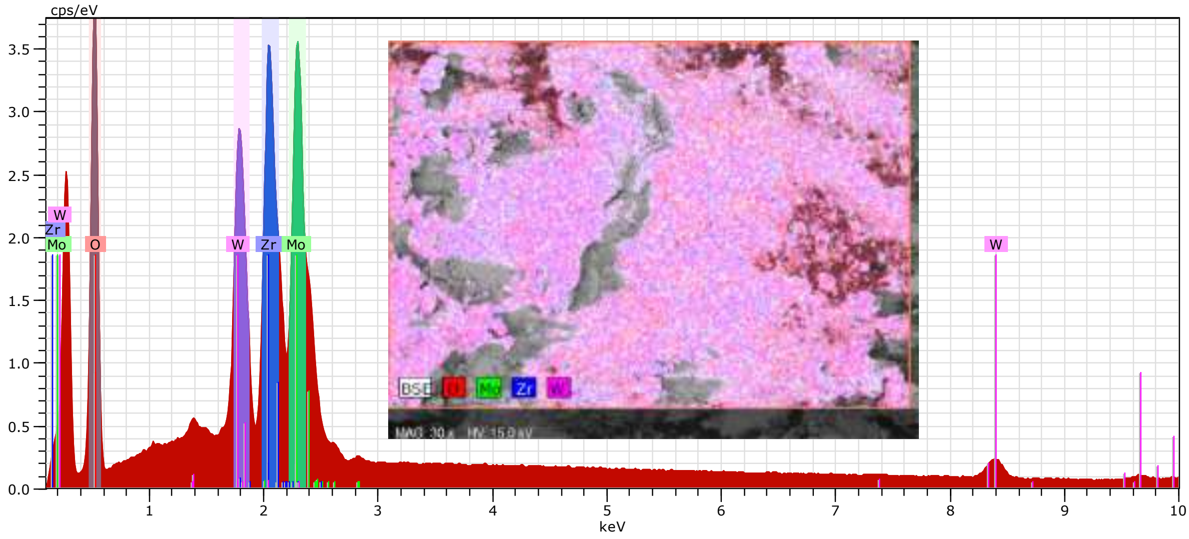

Element	AN	[norm. at.%]	Error in %
Molybdenum	42	77, 4	1,2
Tungsten	74	22,6	0,8
		100	

Point 2.

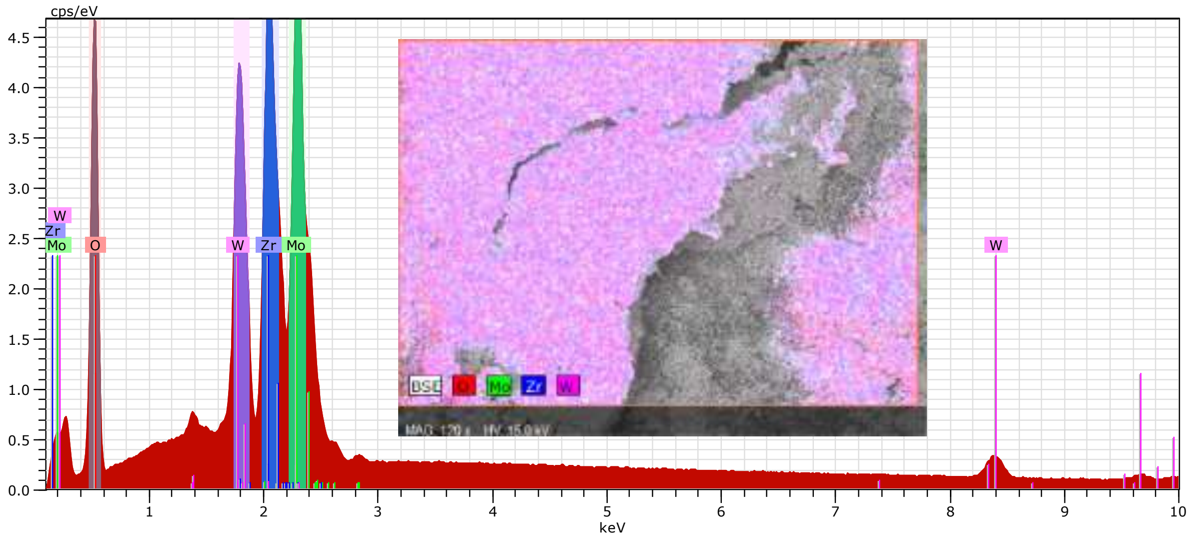

Element	AN	[norm. at.%]	Error in %
Molybdenum	42	77, 2	1,7
Tungsten	74	22,8	0,9
		100	

$ZrMo_xW_{2-x}O7(OH,Cl)_2{\cdot}2H_2O$, x = 1.8

Point 1.

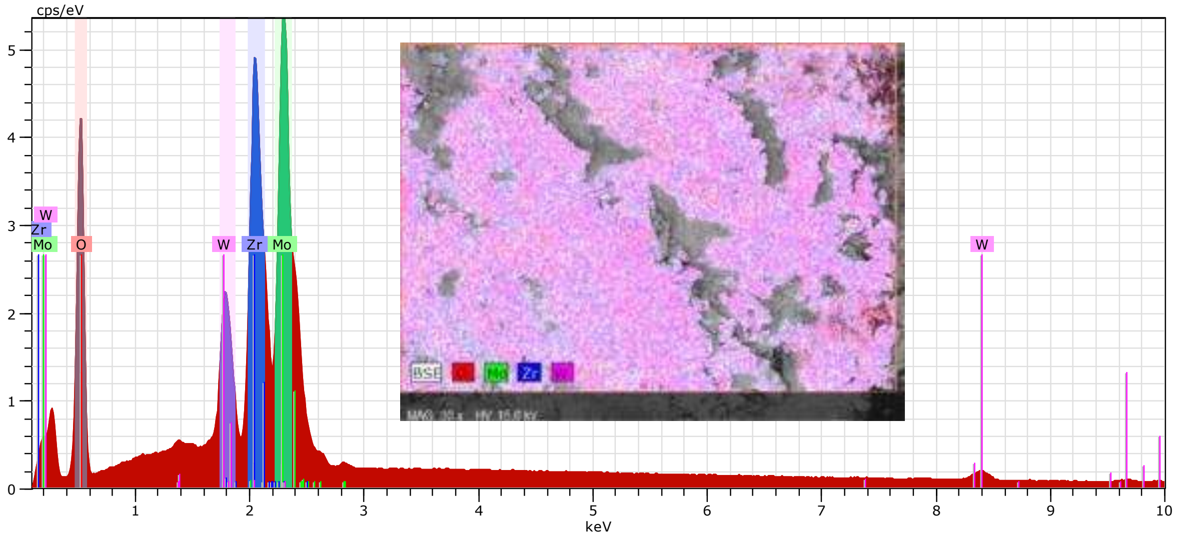

Element	AN	[norm. at.%]	Error in %
Molybdenum	42	87, 3	1,3
Tungsten	74	12,7	0,5
		100	

Point 2.

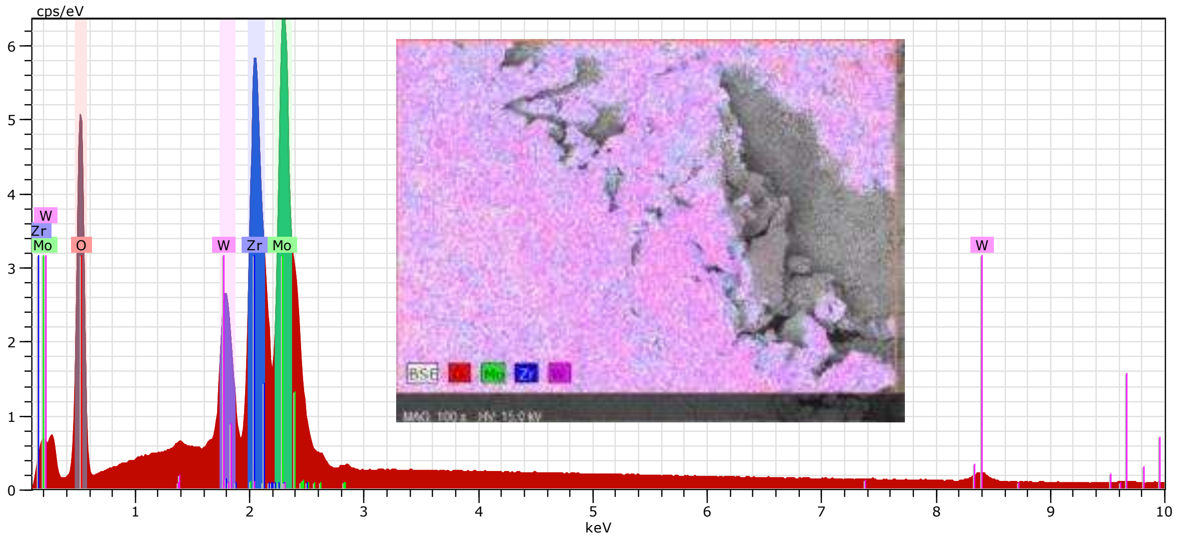

Element	AN	[norm. at.%]	Error in %
Molybdenum	42	87, 3	1,3
Tungsten	74	12,7	0,5
		100	

$ZrMo_xW_{2-x}O7(OH,Cl)_2 \cdot 2H_2O$, $x = 2.0$

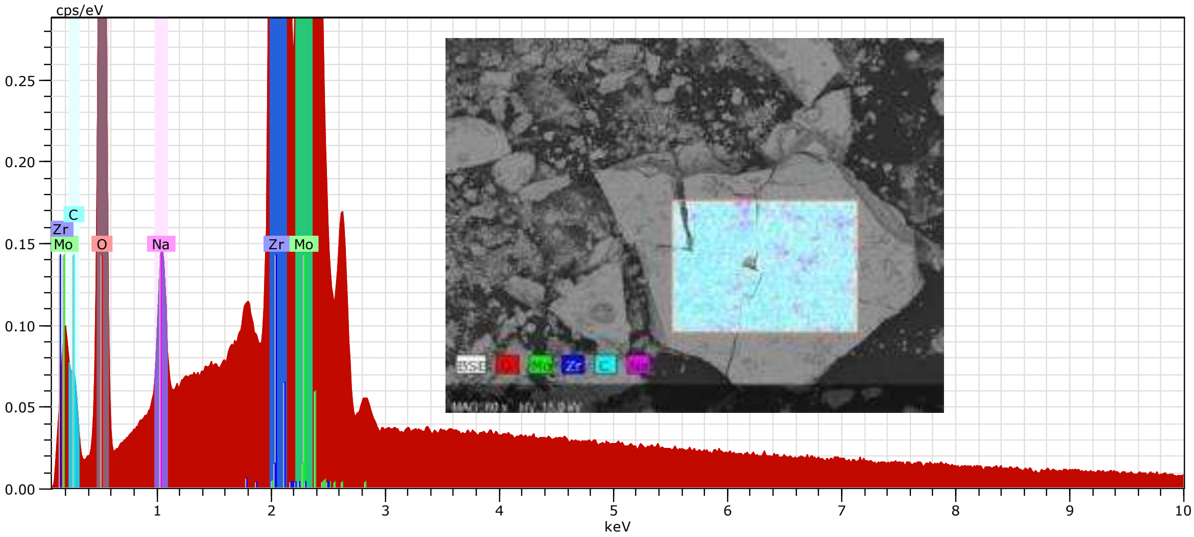

Supplementary Figure 4 | Data of high-temperature PXRD for a family of $ZrMo_xW_{2-x}O_7(OH,Cl)_2 \cdot 2H_2O$ and $ZrMo_xW_{2-x}O_8$.

High-temperature experiments were carried out at the station of time-resolved diffractometry, channel 5b of the Siberian Synchrotron and Therahertz Radiation Centre. Wavelength used was 1.516 A. Diffraction patterns were recorded by a one-coordinate detector OD-3 developed in BINP . Exposure time for a frame was set to 1 minute. Heating of the samples were performed in air up to 1123 K with the rate 10 K/min.

In general, the transformations follow the pattern:

Precursor - orthorhombic phase - cubic phase - trigonal phase - oxides.

The disappearance of the precursor is fixed at a peak of about 24.1^0

The appearance of a rhombic phase is fixed by the appearance of a peak at about 21.2 °.

The disappearance of the rhombic phase is fixed at a peak of about 32.5^0

The appearance and disappearance of the cubic phase is fixed by the appearance of a peak of about 23.5^0.

The appearance and disappearance of the trigonal phase is fixed by the appearance of a peak of about 33.9^0.

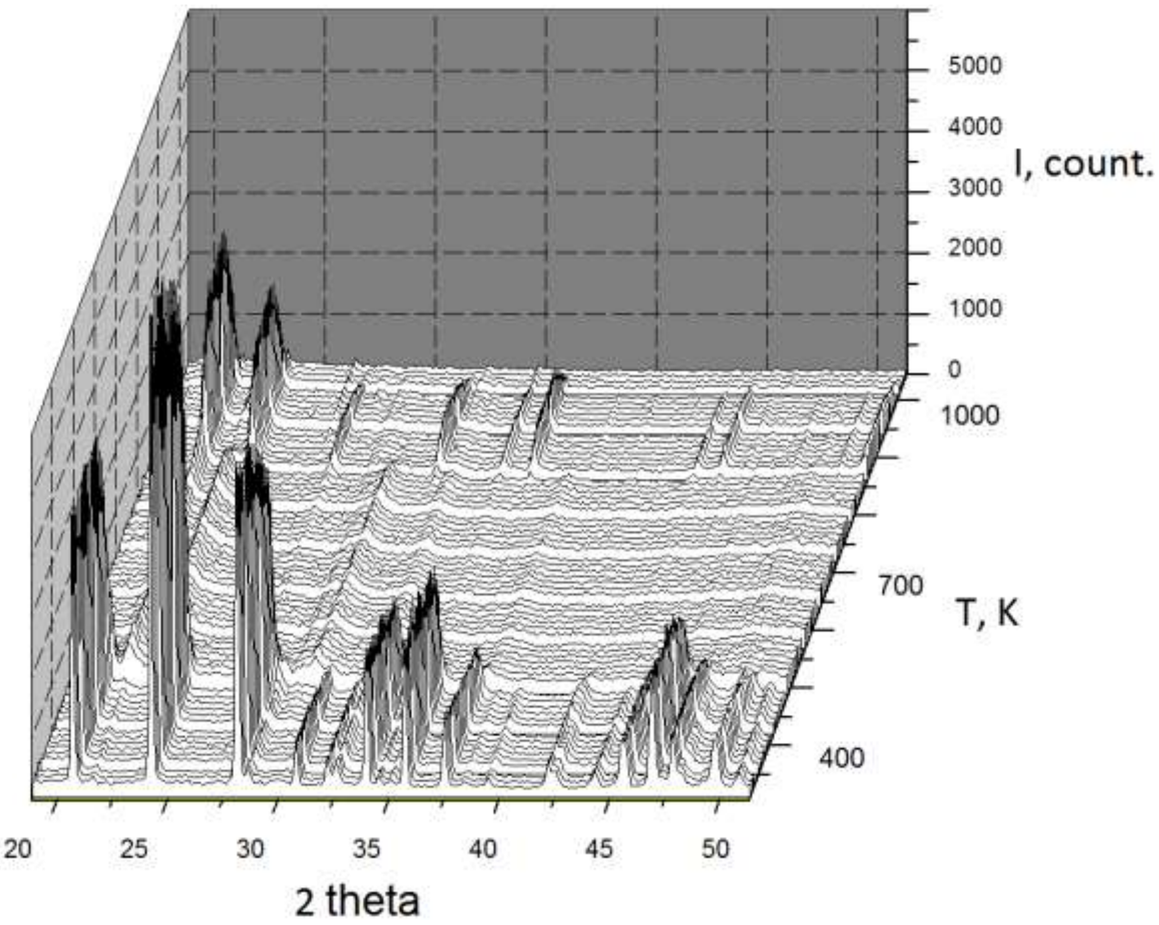

High-temperature PXRD for $ZrMo_xW_{2-x}O_7(OH,Cl)_2{\cdot}2H_2O$ and $ZrMo_xW_{2-x}O_8$ (x = 0.2).

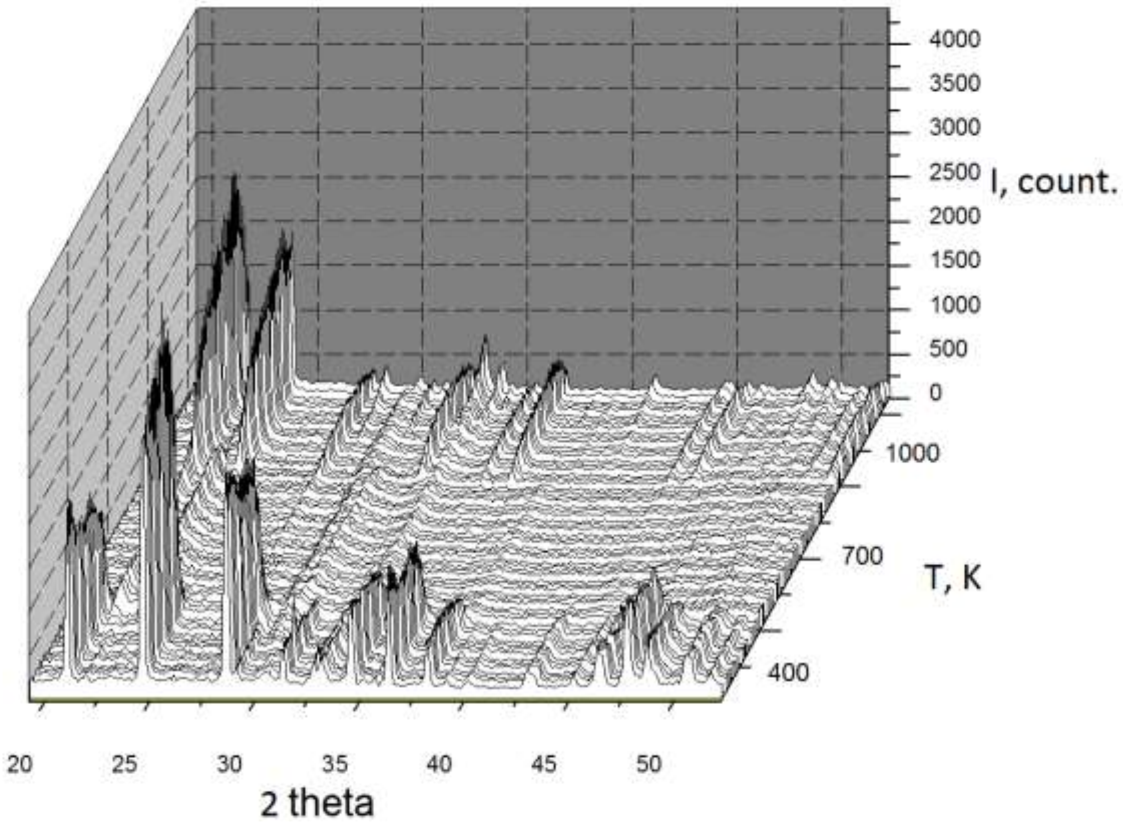

High-temperature PXRD for $ZrMo_xW_{2-x}O_7(OH,Cl)_2{\cdot}2H_2O$ and $ZrMo_xW_{2-x}O_8$ (x = 0.4).

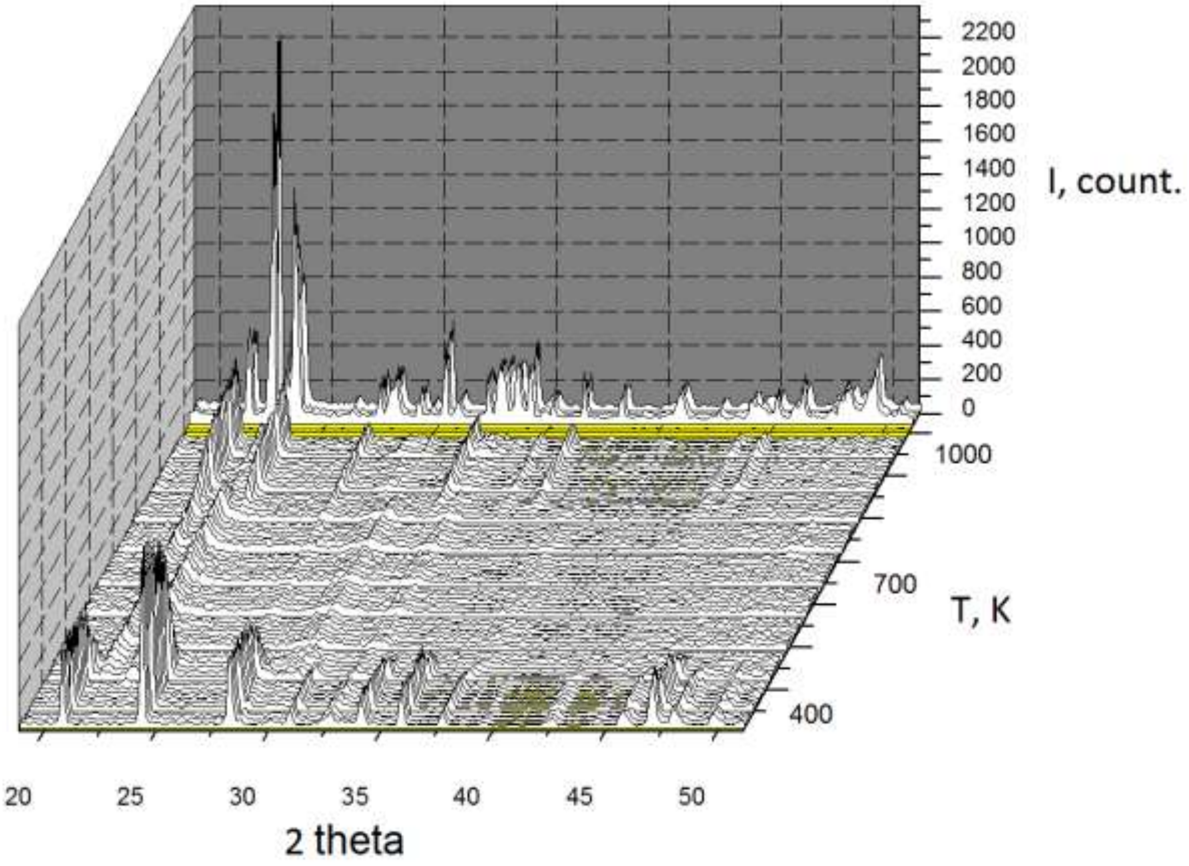

High-temperature PXRD for $ZrMo_xW_{2-x}O_7(OH,Cl)_2\cdot 2H_2O$ and $ZrMo_xW_{2-x}O_8$ (x = 0.6).

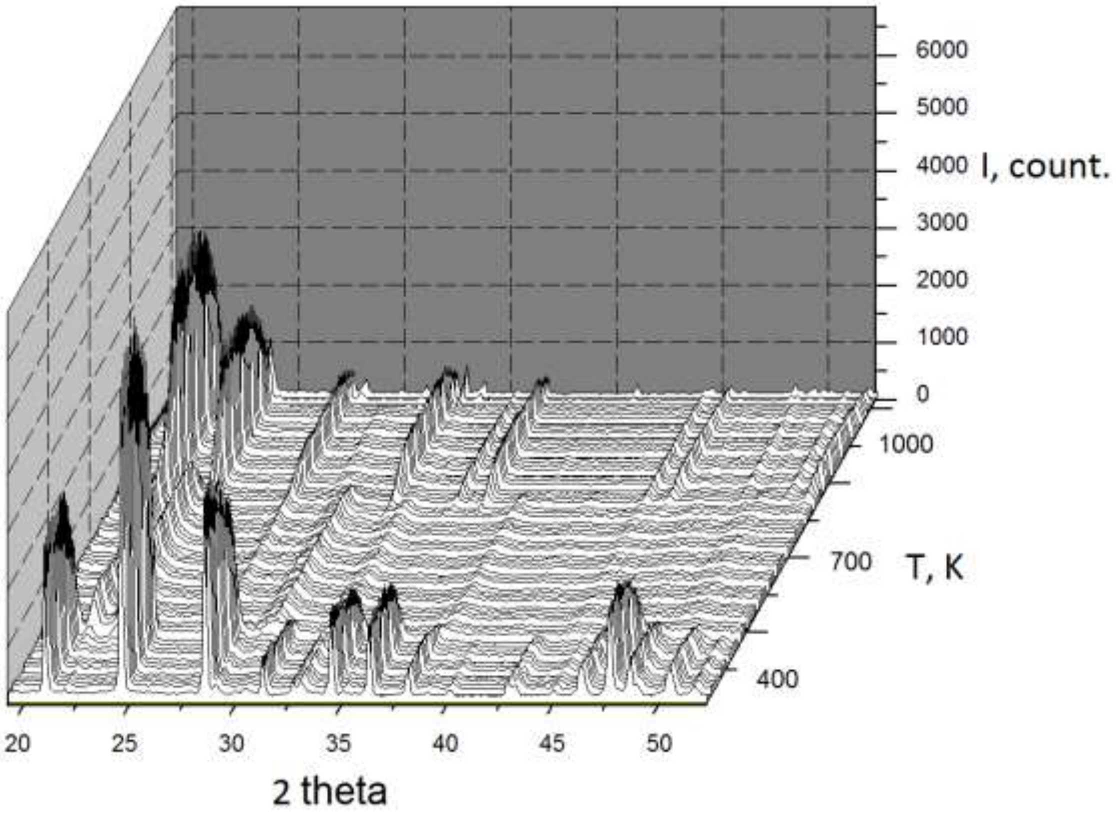

High-temperature PXRD for $ZrMo_xW_{2-x}O_7(OH,Cl)_2\cdot 2H_2O$ and $ZrMo_xW_{2-x}O_8$ (x = 0.8).

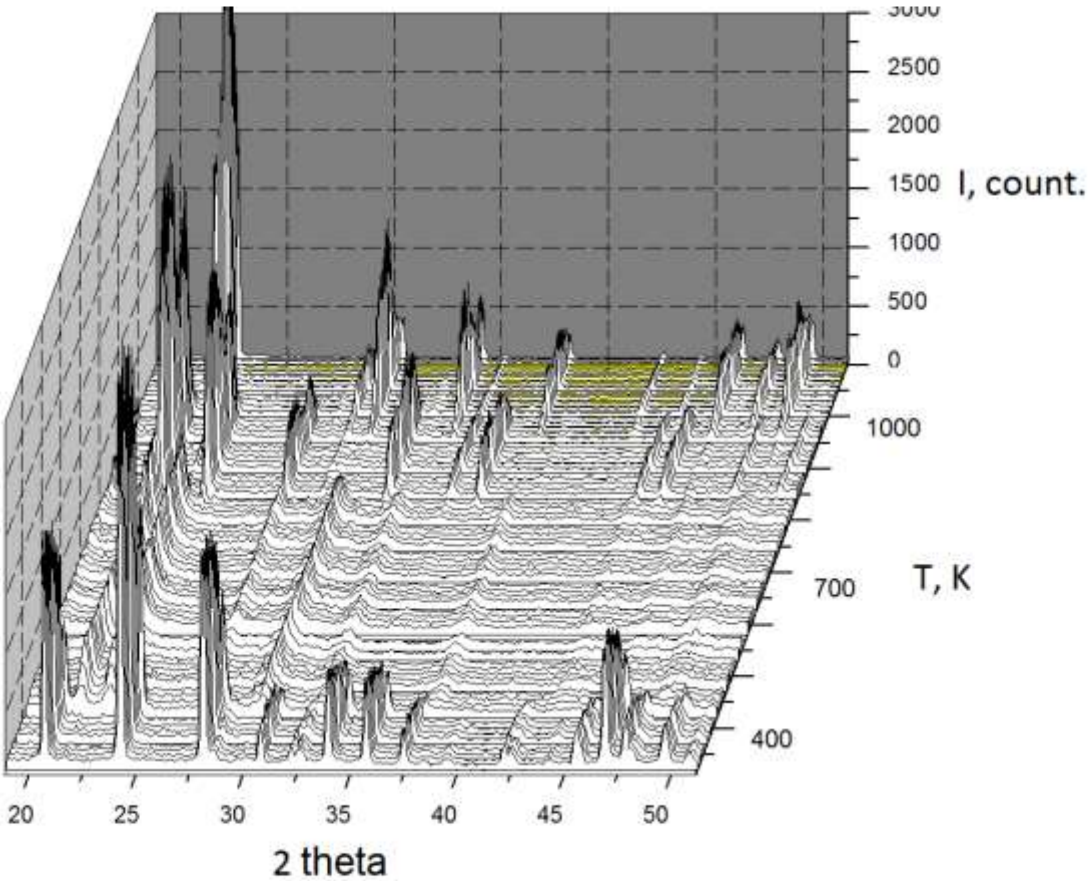

High-temperature PXRD for $ZrMo_xW_{2-x}O_7(OH,Cl)_2 \cdot 2H_2O$ and $ZrMo_xW_{2-x}O_8$ (x = 1.0).

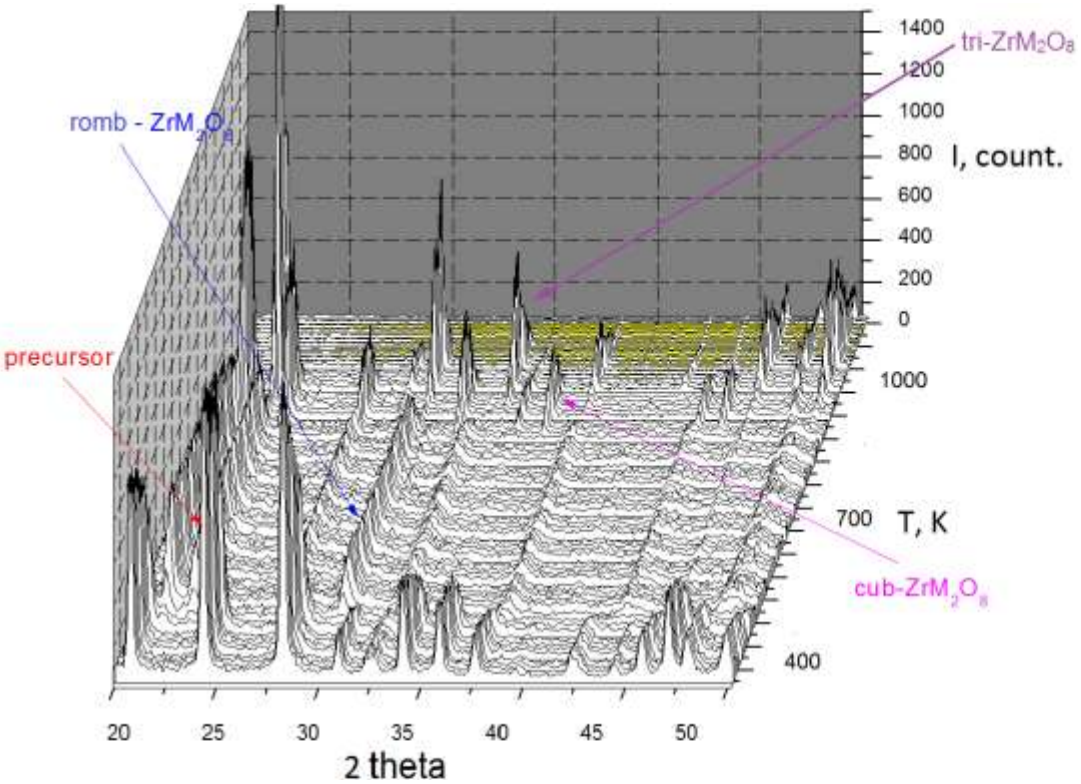

High-temperature PXRD for $ZrMo_xW_{2-x}O_7(OH,Cl)_2 \cdot 2H_2O$ and $ZrMo_xW_{2-x}O_8$ (x = 1.2).

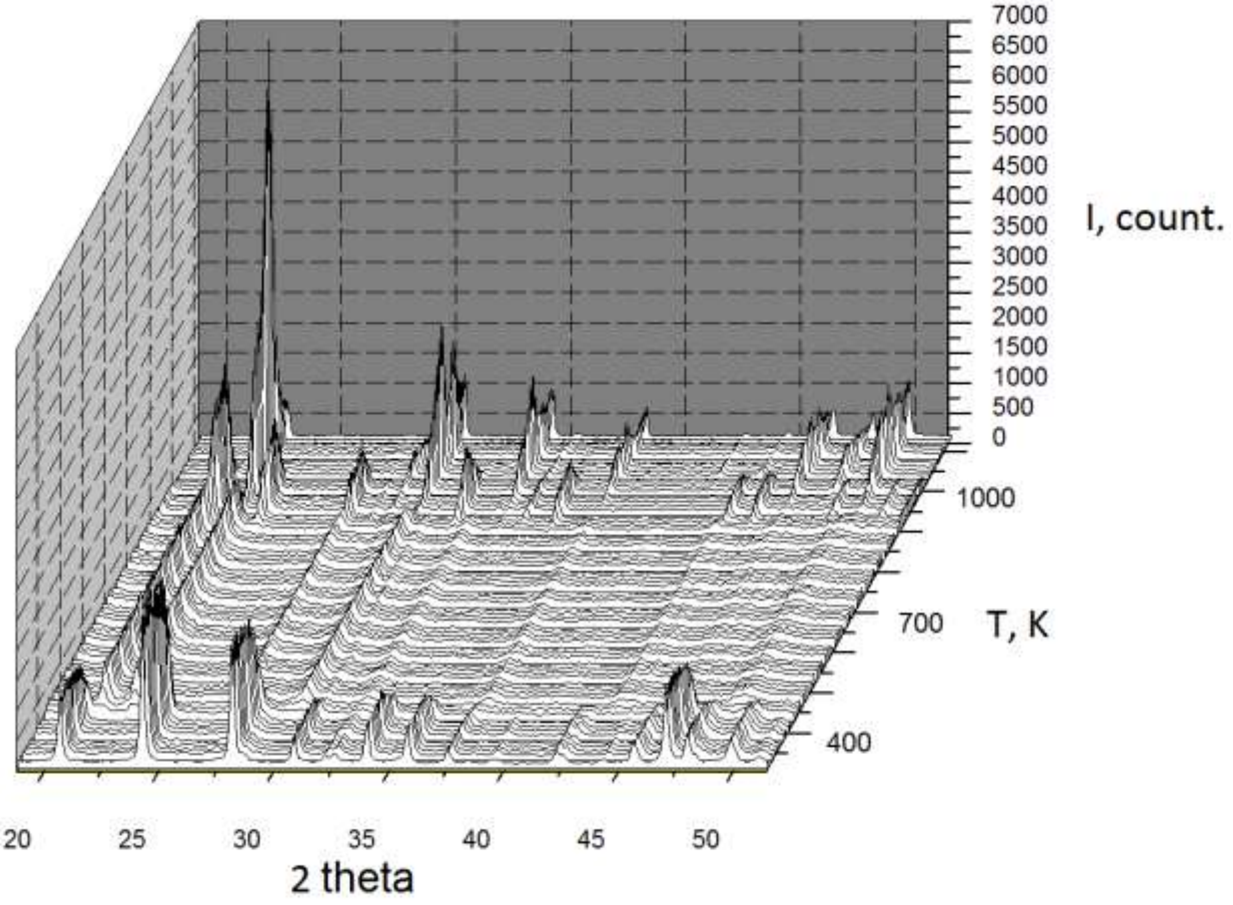

High-temperature PXRD for $ZrMo_xW_{2-x}O_7(OH,Cl)_2{\cdot}2H_2O$ and $ZrMo_xW_{2-x}O_8$ (x = 1.4).

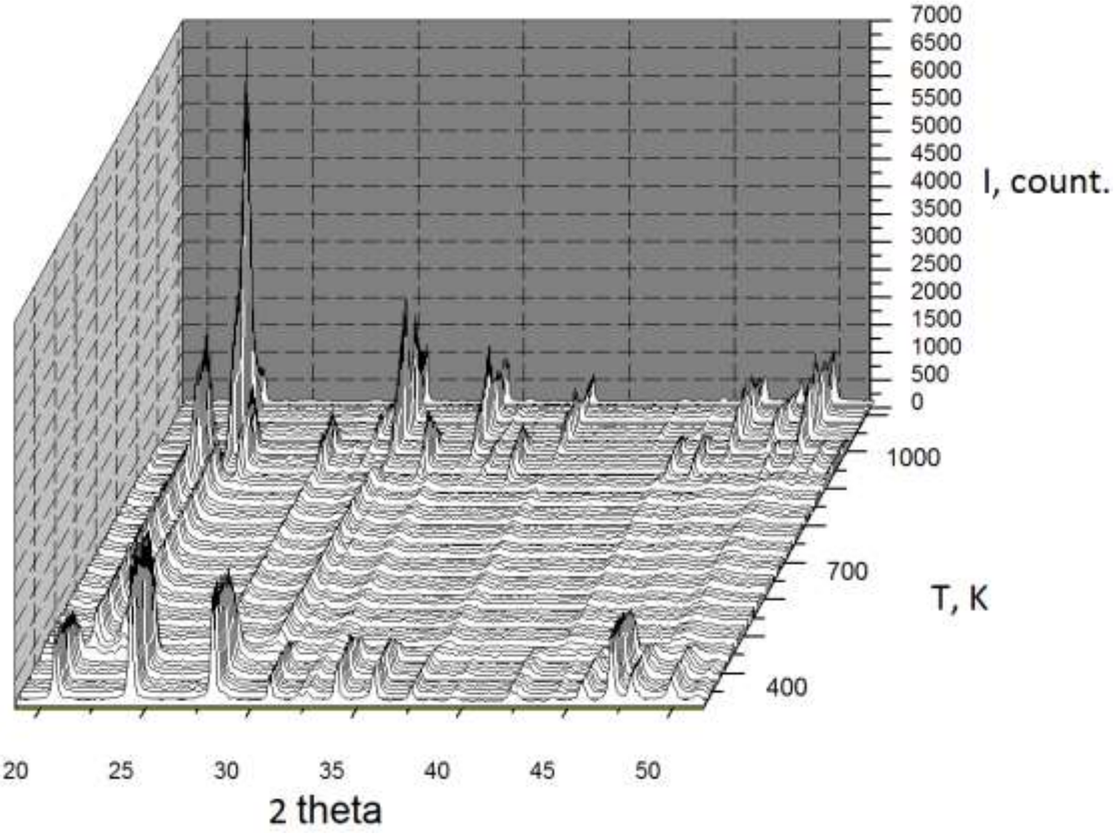

High-temperature PXRD for $ZrMo_xW_{2-x}O_7(OH,Cl)_2{\cdot}2H_2O$ and $ZrMo_xW_{2-x}O_8$ (x = 1.6).

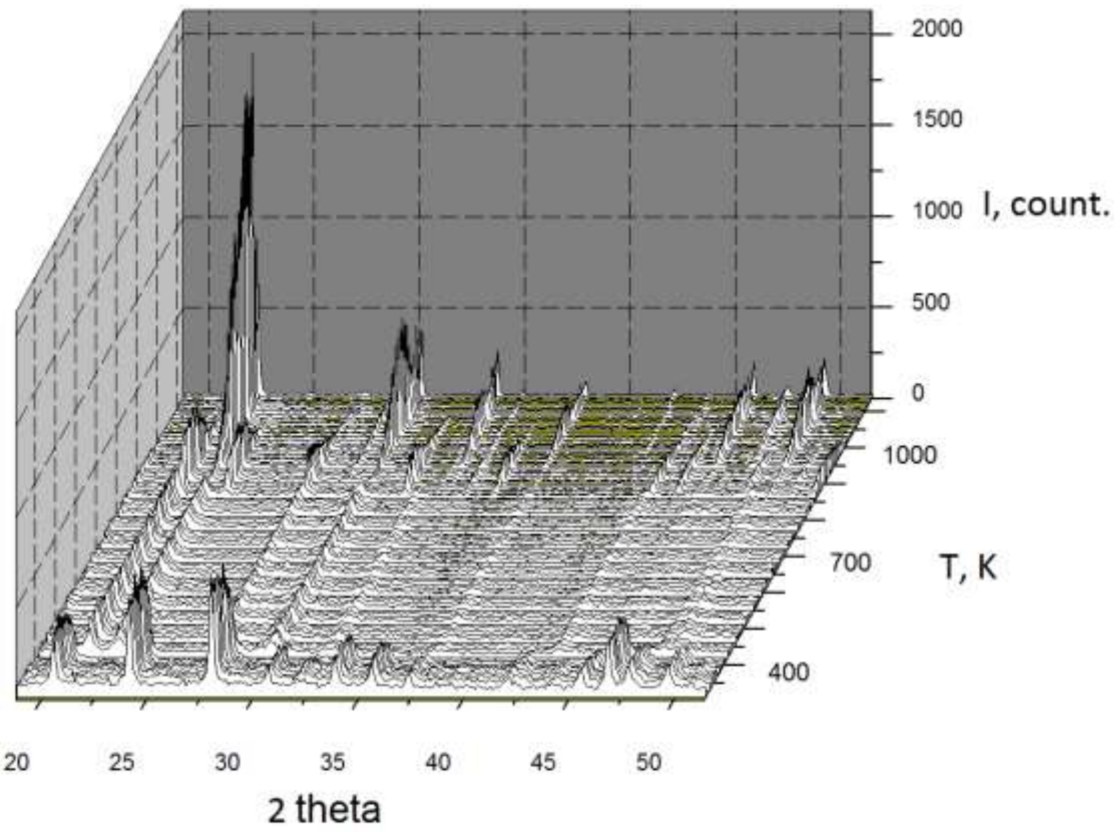

High-temperature PXRD for $ZrMo_xW_{2-x}O_7(OH,Cl)_2{\cdot}2H_2O$ and $ZrMo_xW_{2-x}O_8$ (x = 1.8).

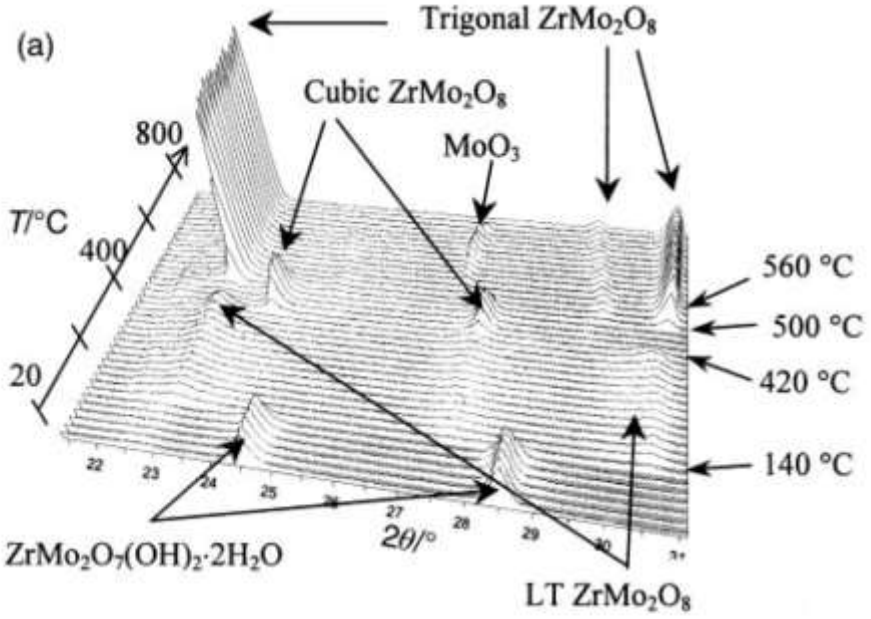

High-temperature PXRD for $ZrMo_xW_{2-x}O_7(OH,Cl)_2{\cdot}2H_2O$ and $ZrMo_xW_{2-x}O_8$ (x = 2.0)*.

* Lind Cora, et al. "Preparation of the negative thermal expansion material cubic $ZrMo_2O_8$." *Journal of Materials Chemistry* 11.12 (2001): 3354-3359.)

Supplementary Table 1 | The lattice parameters and the crystal size of the cubic phase $ZrW_{2-x}Mo_xO_8$.

T, K	a, Å	D, nm	a, Å	D, nm
	x=0.2		x=0.4	
473	9.1327 (6)	9.1278 (9)	9.1278 (9)	60 (1)
523	9.1319 (6)	9.1267 (9)	9.1267 (9)	59 (1)
573	9.1297 (6)	9.1249 (9)	9.1249 (9)	61 (1)
623	9.1278 (6)	9.1230 (9)	9.1230 (9)	60 (2)
673	9.1267 (6)	9.1220 (9)	9.1220 (9)	60 (3)
773	9.1233 (5)	9.1183 (9)	9.1183 (9)	67 (2)
CTE*10^{-6}	-3.51(12)(		-3.50 (13)	
T, K	x=0.6		x=1.0	
473	9.1230 (6)	9.1336 (3)	9.1336 (3)	56 (1)
523	9.1211 (7)	9.1324 (3)	9.1324 (3)	58 (1)
573	9.1200 (7)	9.1296 (2)	9.1296 (2)	63 (1)
623	9.1186 (7)	9.1275 (2)	9.1275 (2)	68 (2)
673	9.1168 (6)	9.1255 (3)	9.1255 (3)	69 (2)
773	9.1133 (6)	9.1218 (2)	9.1218 (2)	72 (2)
CTE*10^{-6}	-3.47(13)		-4.48(11)	
T, K	x=1.2		x=1.4	
373	9.1329 (2)	52 (1)	9.1371 (4)	58.9 (8)
473	9.1288 (2)	53 (1)	9.1328 (3)	60 (1)
523	9.1262 (3)	54 (1)	9.1313 (3)	59 (1)
573	9.1240 (2)	55 (1)	9.1302 (3)	61 (1)
623	9.1220 (2)	55 (1)	9.1273 (3)	60 (2)
673	9.1203 (2)	56 (1)	9.1258 (4)	60 (3)
773	9.1168 (2)	56 (1)	9.1224 (3)	67 (2)
CTE*10^{-6}	-4.47(14)		-4.00(14)	
T, K	x=1.6			
303	9.1380 (4)	65 (3)		

373	9.1342 (4)	77 (4)
473	9.1297 (4)	74 (3)
523	9.1278 (4)	75 (4)
573	9.1259 (4)	72 (3)
623	9.1235 (4)	75 (3)
673	9.1223 (4)	72 (3)
773	9.1188 (5)	71 (4)
CTE*10^{-6}	-4.49(16)	
T, K	x=1.8	
298	9.13745(4)	113.8(12)
323	9.13481(5)	113.8(12)
373	9.13078(7)	113.8(12)
423	9.12814(7)	113.8(12)
473	9.12592(7)	113.8(12)
523	9.12382(7)	113.8(12)
573	9.12218(6)	113.8(12)
623	9.12036(6)	113.8(12)
673	9.11882(6)	113.8(12)
723	9.11733(6)	113.8(12)
773	9.11604(6)	113.8(12)
CTE*10^{-6}	-9.6(2) /298-373 K<br>-3.98(8) /373-773 K	
T, K	x=0.8	
341	9.11135(50)	47.47(58)
353	9.10732(48)	46.11(53)
364	9.10732(47)	51.05(62)
374	9.10677(46)	56.36(73)
384	9.10551(47)	57.32(76)
394	9.10594(47)	57.38(76)

404	9.10575(47)	56.95(76)
414	9.10544(46)	57.92(77)
423	9.10442(47)	55.72(74)
454	9.10354(46)	56.68(75)
483	9.10322(46)	57.99(78)
515	9.10245(46)	58.53(80)
543	9.10243(46)	58.75(80)
577	9.10075(48)	58.45(81)
604	9.09925(47)	58.94(81)
635	9.09890(47)	58.68(81)
664	9.09763(47)	58.67(81)
693	9.09666(47)	59.28(83)
724	9.09527(47)	59.15(83)
757	9.09726(48)	59.43(84)
784	9.09318(48)	59.04(83)
818	9.09161(48)	59.30(85)
843	9.09093(51)	58.50(85)
878	9.09093(51)	58.50(85)
907	9.09017(52)	58.21(84)
CTE*10^{-6}	-3.55(16)	
